# Genetically engineered HEK cells as a valuable tool for studying electroporation in excitable cells

**DOI:** 10.1038/s41598-023-51073-5

**Published:** 2024-01-06

**Authors:** Tina Batista Napotnik, Bor Kos, Tomaž Jarm, Damijan Miklavčič, Rodney P. O’Connor, Lea Rems

**Affiliations:** 1 University of Ljubljana, Faculty of Electrical Engineering, Tržaška Cesta 25, 1000 Ljubljana, Slovenia; 2 École des Mines de Saint-Étienne, Department of Bioelectronics, Georges Charpak Campus, Centre Microélectronique de Provence, 880 Route de Mimet, 13120 Gardanne, France

**Keywords:** Membrane biophysics, Neurophysiology, Computational models

## Abstract

Electric pulses used in electroporation-based treatments have been shown to affect the excitability of muscle and neuronal cells. However, understanding the interplay between electroporation and electrophysiological response of excitable cells is complex, since both ion channel gating and electroporation depend on dynamic changes in the transmembrane voltage (TMV). In this study, a genetically engineered human embryonic kidney cells expressing Na_V_1.5 and K_ir_2.1, a minimal complementary channels required for excitability (named S-HEK), was characterized as a simple cell model used for studying the effects of electroporation in excitable cells. S-HEK cells and their non-excitable counterparts (NS-HEK) were exposed to 100 µs pulses of increasing electric field strength. Changes in TMV, plasma membrane permeability, and intracellular Ca^2+^ were monitored with fluorescence microscopy. We found that a very mild electroporation, undetectable with the classical propidium assay but associated with a transient increase in intracellular Ca^2+^, can already have a profound effect on excitability close to the electrostimulation threshold, as corroborated by multiscale computational modelling. These results are of great relevance for understanding the effects of pulse delivery on cell excitability observed in context of the rapidly developing cardiac pulsed field ablation as well as other electroporation-based treatments in excitable tissues.

## Introduction

High-intensity electric pulses cause a transient increase in cell plasma membrane permeability for otherwise poorly permeant molecules and the phenomenon is termed electroporation. Electroporation-based techniques are increasingly used in numerous applications in medicine^1–4^, food technology^5,6^, and biotechnology^7^.

In many electroporation-based medical treatments the excitable cells (muscle and nerve cells) are affected by the electric pulses even when they are not the primary target of the treatment^8–10^. This can lead to undesirable side effects such as pain and muscle contraction^11,12^ or nerve damage^13,14^. In a promising new application—pulsed‐field ablation which utilizes irreversible electroporation for treating heart arrhythmias^3,15^—it is not yet understood how reversibly electroporated cells on the borders of treated area respond to the treatment and how this response affects the functioning of the heart after the treatment^16^. The disappearance of the local intracardiac electrograms immediately after pulse delivery does not necessarily indicate that a permanent lesion has been created^17^, which points to some transient effects of electric pulses on cardiac cells that still need to be elucidated. Therefore, it is of great importance to study how excitable cells respond to electric pulses used for electroporation.

Excitable cells can generate and propagate electrical signals called action potentials (APs), where voltage-gated ion channels that open and close in response to small changes in transmembrane voltage (TMV) play an essential role. It has been long known that external electric fields can activate voltage-gated ion channels and trigger APs, and this electrostimulation is nowadays used for various purposes (e.g. implantable cardiac pacemakers, heart defibrillation, and deep brain stimulation)^18–20^. However, when the plasma membrane becomes electroporated, additional ionic current passes through pores/defects created in the membrane by the electric field, which can affect cell excitability^21,22^. The interplay between excitation and electroporation is complex, since both ion channel gating and creation of pores/defects depend on dynamic changes in the TMV.

To understand the effects of electric pulses on excitable cells, in vitro experiments are of the utmost importance. However, there are several drawbacks in using isolated primary excitable cells, such as primary cardiomyocytes or neurons: their isolation from animals must be ethically approved; the procedure is time consuming and requires great technical skills. Furthermore, isolated cardiomyocytes deteriorate and lose their characteristic behaviour very fast (within a few hours to days)^23^, they do not multiply and can be overgrown by non-cardiomyocyte cells, the possibilities of genetic manipulations are limited^24–26^. One alternative to primary cardiomyocytes are cardiac cell lines, such as the rat cardiomyoblast cell line H9c2^27^, the mouse atrial myocyte-derived HL-1 line^28^ and the human AC16 line^29^, which are easy to propagate and maintain and allow high-throughput screening. However, they all exhibit a loss of certain cardiomyocyte features such as contractility and robust generation of APs. Another alternative to primary cardiomyocytes are embryonic or induced pluripotent stem cell-derived cardiomyocytes that have some advantages over the aforementioned cell lines but are for now still of limited commercial availability^24–26^.

With the development of synthetic biology, a new possibility to design minimal systems that recreate aspects of naturally evolved variants has emerged. For this study, we used a genetically engineered human embryonic kidney (HEK) cell line that expresses Na_V_1.5 and K_ir_2.1, a minimal complement of sodium and potassium channels required for cell excitability^30–32^. Na_V_1.5 is the predominant cardiac sodium channel subtype responsible for AP initiation^33,34^, and K_ir_2.1 is an inward-rectifier potassium channel expressed in multiple cell types, including cardiomyocytes, where it contributes a significant repolarizing current during the terminal phase of the AP and controls the resting TMV^35–37^. The expression of K_ir_2.1 in these cells is controlled by a doxycycline-induced tet-on system^32^ that allows culturing two cell variants: excitable “spiking” S-HEK (containing Na_V_1.5 and K_ir_2.1) and non-excitable “non-spiking” NS-HEK cells (containing only Na_V_1.5 channels). Intercellular electrical coupling in the cell monolayer is mediated by connexin 43 and 45 gap junctions which are endogenously expressed in HEK cells^38–40^.

The fact that these cells express two known channel subtypes facilitates dramatically the analysis in comparison to other excitable cells, which express multiple different types and subtypes of voltage-gated ion channels, as well as other ion channels that participate in regulating the TMV. Furthermore, due to the doxycycline-induced expression of K_ir_2.1 we can compare the effects of electric fields on the excitable and non-excitable variant of the same cells.

The aim of this study was to characterize the excitable S-HEK cells and their non-excitable counterparts NS-HEK as a model system for studying the effects of electric pulses on AP generation and other changes in TMV. As a first step we studied the response to electric pulses with duration of 100 µs, which are conventionally used in various electroporation-based treatments such as electrochemotherapy^41^ and irreversible electroporation^42^. We found that AP generation in S-HEK cells becomes affected by electroporation already at low electric fields that are close to the electrostimulation threshold, resulting in generation of multiple APs, AP prolongation and, at higher electric fields, sustained membrane depolarization. We developed computational models, both at the microscopic level of individual cells and at the level of the entire cell monolayer, that demonstrate how a small increase in membrane conductance due to electroporation can have profound effects on cell excitability. These results are of great relevance for understanding the effects on cell excitability observed in context of the rapidly developing electroporation-based treatments of the heart and other excitable tissues and organs^9,16,43,44^.

## Results

### S-HEK cells generate action potentials and NS-HEK do not

**Figure 1:**
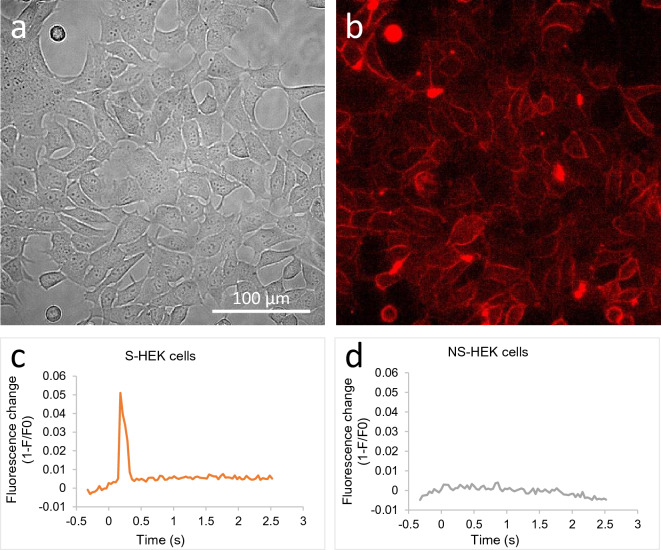
S-HEK cells are able to generate APs whereas NS-HEK are not. (**a**,**b**) A monolayer of S-HEK (spiking HEK) cells, a brightfield (**a**) and a fluorescence image (**b**), the membranes are labelled with the ElectroFluor630 potentiometric probe. (**c**,**d**) A single 100 µs, 150 V/cm electric pulse can trigger AP (as determined by a membrane fluorescence signal from ElectroFluor630, combined from all the cells in the field of view) in S-HEK cells (**c**) but not in NS-HEK cells (**d**). Pulse was delivered at time zero. There is a delay between the pulse delivery and the onset of AP in Fc, which is analysed further in Fig. 3f.

S-HEK cells (Fig. 1a,b) that were genetically modified to constitutively express Na_V_1.5 channels and with a doxycycline-induced expression of K_ir_2.1 channels were able to generate APs, as detected with a fluorescent potentiometric probe ElectroFluor630 (Fig. 1c). NS-HEK cells that were not incubated with doxycycline and did not express K_ir_2.1 channels, only Na_V_1.5 channels, were not able to generate APs (Fig. 1d).

### The effect of electric pulses on transmembrane voltage response in S-HEK and NS-HEK cells

**Figure 2:**
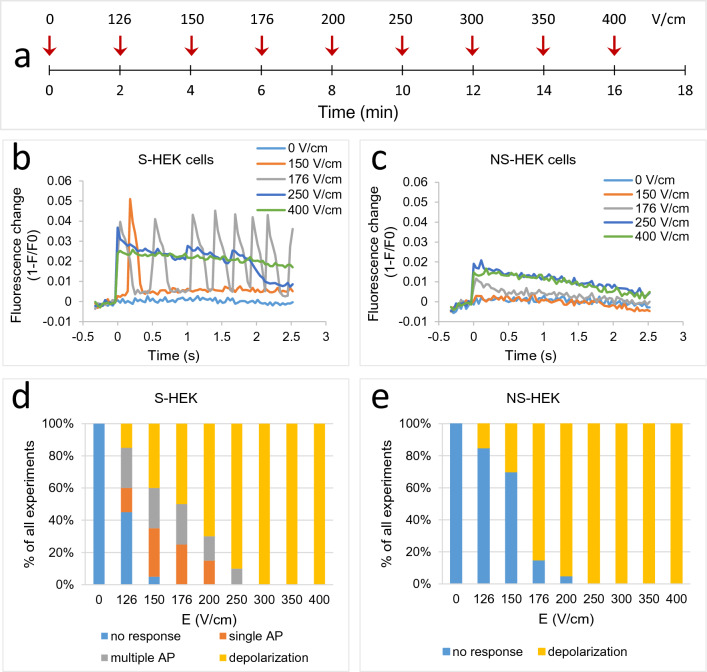
(**a**) Experiment timeline. Each sample was exposed to a sequence of eight consecutive 100 µs electric pulses of different electric field strength (E ≈ 126–400 V/cm) applied 2 min apart. The arrows represent each pulse application. (**b**,**c**) Triggering changes in TMV in excitable S-HEK (**b**) and non-excitable NS-HEK cells (**c**) with the pulse sequence (results from a representative sample, only TMVs after selected E are shown, ElectroFluor630 fluorescence signal was combined from all the cells in the field of view). Pulses were delivered at time zero. (**d**,**e**) The experiments with different responses to electric pulses in S-HEK (**d**) and NS-HEK (**e**) were counted and expressed as % of all experiments. Number of experiments: N = 20 for both cell variants.

We exposed excitable S-HEK and non-excitable NS-HEK cells to a sequence of eight consecutive 100 µs pulses with increasing electric field (E ≈ 126–400 V/cm), applied 2 min apart (Fig. 2a). The 2 min interval was chosen as a compromise between the pulses being as far apart as possible and the cells not being exposed to ambient conditions for too long (one experiment lasted up to one hour, including cell labelling). Time-lapse images were acquired for each pulse (80 images with 36 ms interval, a pulse was applied after 10th image). The analysis of ElectroFluor630 fluorescence revealed that at the lower electric fields S-HEK generated one or multiple APs (Fig. 2b,d), whereas NS-HEK responded only with a smaller change in TMV (Fig. 2c,e). As can be seen from Fig. 2d, the responses vary at the same E, particularly at lower E. At higher electric fields (≥ 250 V/cm), APs in S-HEK cells gradually became longer and needed more time to recover resulting in a sustained depolarization. Eventually, the TMV in S-HEK cells did not return to the baseline in 2.5 s after pulse exposure (Fig. 2b, 250 and 400 V/cm). We separated the responses in S-HEK cells to a single AP, multiple APs, and a sustained depolarization (Fig. 2d). The duration of the sustained depolarization increased with the increasing electric field (Fig. 2b, 250 and 400 V/cm). In NS-HEK cells, we observed a similar progressively longer sustained depolarization at higher electric fields, however, without any APs (Fig. 2c, 250 and 400 V/cm, and Fig. 2e).

We further analysed ElectroFluor630 fluorescence signals of S-HEK and NS-HEK cells and determined the following parameters for each pulse exposure: the number of peaks, the peak amplitude (maximal TMV response), time to first peak, and time to 25%, 50% and 90% recovery to the baseline. In the following paragraphs we present a detailed analysis of all characteristic responses and their dependence on the applied E.

In S-HEK cells, multiple APs (two or more) were triggered at lower E (126–250 V/cm), e.g., appearing at 126 V/cm in almost half of experiments with a response (Fig. 2d). The occurrence of multiple APs diminished with increasing E, and the response gradually became a sustained depolarization, not AP (see Fig. 2b, 250 and 400 V/cm). When the response was multiple APs, electric pulses triggered from 2 to 9 APs (with an average of around four APs, Supplement 1, Fig. S1) within 2.5 s after pulse delivery, but the spontaneous APs could continue further (see Fig. 2b, 176 V/cm).

**Figure 3:**
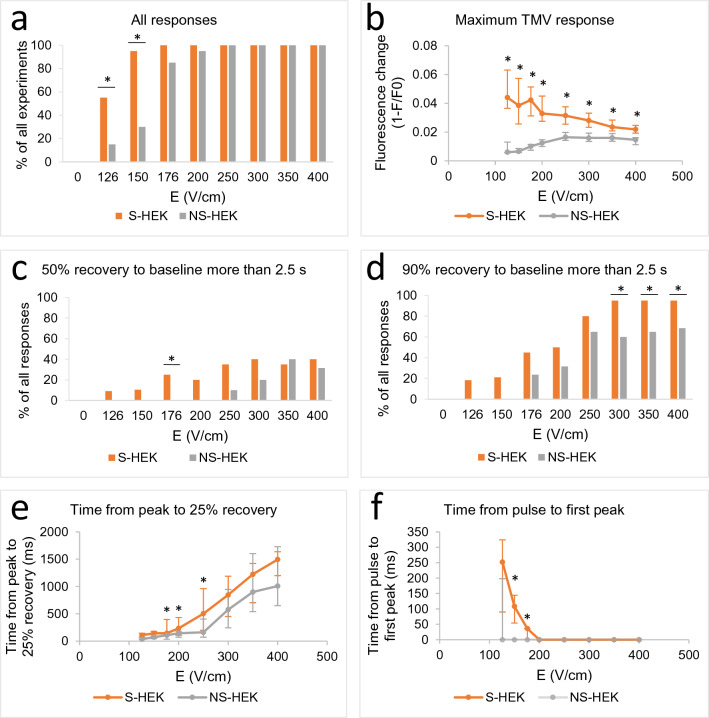
The parameters of TMV responses triggered by the pulse sequence shown in Fig. 2a in excitable S-HEK and non-excitable NS-HEK cells. In (**a**), all the experiments with responses are counted and expressed as % of all experiments. In (**b**), the amplitude of TMV responses. In (**c**–**e**), the recovery of TMV is shown: the percentage of responses where 50% (**c**) or 90% (**d**) of recovery towards the baseline value was reached later than 2.5 s after the pulse (the end of observation time). (**e**) Time from the first peak to 25% recovery. (**f**) Time from pulse exposure to first peak. The results are expressed as medians with bars Q1 and Q3. Number of experiments: N = 20 for both cell variants. *Statistical difference between S-HEK and NS-HEK cells (p < 0.05), Fisher’s Exact test (**a**,**c**,**d**), Mann–Whitney Rank Sum Test (**b**,**e**,**f**).

The threshold for AP in S-HEK cells was somewhat lower than for changes in TMV in NS-HEK cells: significantly more responses at low E (126 and 150 V/cm) were evoked in S-HEK cells than in NS-HEK (Fig. 3a). The amplitude of an AP was much higher than the change in TMV of NS-HEK (Fig. 3b). However, as E increased and both S-HEK and NS-HEK exhibited sustained depolarization, the maximum response of S-HEK cells approached that of NS-HEK cells. Note that NS-HEK cells have a lower resting voltage (− 20 mV) compared to S-HEK cells (< − 70 mV)^31,45^ and can thus depolarize to a lesser extent.

Since responses were observed already at the lowest E (126 V/cm), we tested the responses at even lower E in separate experiments where cells were exposed to a sequence of pulses of E ≈ 40, 66, and 100 V/cm. A few responses were observed at these low electric fields, and here too, excitable S-HEK cells responded at lower E than NS-HEK. However, the results of S-HEK and NS-HEK cells were not significantly different (Supplement 1, Table S1). S-HEK responded with single or multiple APs, whereas NS-HEK with a short depolarization.

Recovery of the TMV required more time in S-HEK cells: the percentage of experiments where the signal did not reach 50% or 90% recovery towards the baseline in 2.5 s after pulse exposure (during the observation time) was higher in S-HEK cells than in NS-HEK cells (Fig. 3c,d). Time from the first peak to 25% recovery towards the baseline increased with increasing E and was significantly longer in S-HEK than in NS-HEK cells (176–250 V/cm, see Fig. 3e). The 50% or 90% recovery times could not be determined since they lasted longer than the observation time at higher E in the majority of experiments (Fig. 3c,d). Longer recovery time in S-HEK cells may be due to the higher maximum TMV response in S-HEK cells compared to NS-HEK.

We observed that at lower E, the first AP in S-HEK cells occurred after a short delay (Fig. 3f, see also Fig. 2b). This delay was around 250 ms at the lowest E and gradually decreased with increasing E. At E ≥ 200 V/cm, the change in TMV (AP or sustained depolarization) occurred immediately after pulse exposure (within 36 ms, which was our time resolution). A short delay occurred also in some responses of NS-HEK cells; however, the delay was significantly shorter than in S-HEK.

### Electroporation in S-HEK and NS-HEK cells as determined by propidium uptake

**Figure 4:**
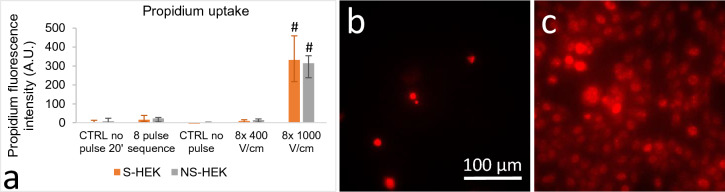
Propidium uptake in S-HEK and NS-HEK cells after electric pulse exposure. Cells were either exposed to the pulse sequence shown in Fig. 2a, or a train of 8 × 100 µs pulses of 400 or 1000 V/cm, 1 Hz. (**a**) Propidium uptake was evaluated as the propidium fluorescence intensity in the nuclear area, background was subtracted. Two controls were used, one after 20 min and one after 5 min of propidium incubation, that served as control for the pulse sequence and pulse train, respectively. The results are expressed as medians with bars Q1 and Q3. Number of experiments: N = 6 for the train of pulses and N = 7 for the sequence. # show significant difference from control, One way ANOVA on ranks. (**b**,**c**) Representative propidium fluorescence images of excitable S-HEK cells after being exposed to the whole pulse sequence shown in Fig. 2a (**b**) or 8 × 100 µs, 1000 V/cm, 1 Hz (**c**).

We used propidium uptake as a standard method for electroporation detection. The complete sequence of all eight 100 µs electric pulses of increasing E (as used in ElectroFluor630 experiments) caused a very weak increase in plasma membrane permeability detected by propidium uptake, however, the results were not significantly different from control (Fig. 4). In contrast, we detected a pronounced propidium uptake after pulses typically used in electrochemotherapy (8 × 100 µs, 1000 V/cm, 1 Hz). A train of 8 × 100 µs, 400 V/cm, chosen as eight pulses of the highest electric field from ElectroFluor630 experiments, delivered in a train with 1 Hz repetition rate, led to a very faint propidium labelling, however, the results were not significantly different from control. No difference was observed between S-HEK and NS-HEK cells.

The morphology of cells was affected only after 8 × 100 µs, 1000 V/cm, 1 Hz pulses (see Supplement 1, Fig.  S2): cells were blebbing, rounding, and detaching, nuclei became condensed and brightly stained with propidium. The morphology of cells treated with other pulse protocols appeared normal, however, in some cells very faint labelling of the whole cell with propidium was seen (but nevertheless, propidium fluorescence in the nuclear area was not significantly different from control, see Fig. 4).

### The effect of electric pulses on calcium response in S-HEK and NS-HEK cells

**Figure 5:**
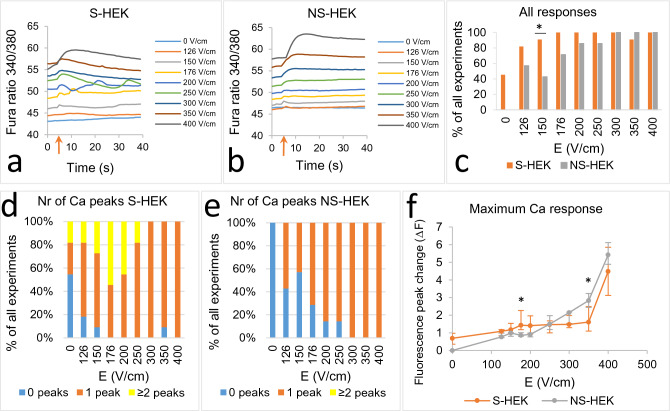
Ca^2+^ response in S-HEK and NS-HEK to the pulse sequence shown in Fig. 2a. (**a**,**b**) Uptake of Ca^2+^ ions in S-HEK (**a**) and NS-HEK cells (**b**) exposed to the pulse sequence (results from a representative batch of cells), as determined by Fura-2 ratio 340/380. Fura-2 ratio was averaged over each whole image. A pulse was delivered at around 5th second after the image acquisition started (red arrow). In (**c**), all the experiments with responses are counted and expressed as % of all experiments. (**d**,**e**) Ca^2+^ response in S-HEK and NS-HEK exposed to the pulse sequence. Number of experiments with 0, 1 or more Ca^2+^ peaks in S-HEK (**d**) and NS-HEK (**e**) are counted and expressed as % of all experiments. (**f**) The amplitude of Ca^2+^ responses triggered by individual pulses of the pulse sequence. The results in (**f**) are expressed as medians with bars Q1 and Q3. Number of experiments: N = 11 for S-HEK cells and N = 7 for NS-HEK cells. *Statistical difference between S-HEK and NS-HEK cells (p < 0.05), Fisher’s Exact test (**c**), and Mann–Whitney Rank Sum Test (**f**).

In addition to TMV response and propidium uptake, we also monitored intracellular Ca^2+^ using ratiometric imaging of the Fura-2 dye, as a surrogate measure of electroporation (Fig. 5). We must be aware however that this Ca^2+^ response can include the entry of Ca^2+^ ions through pores in the membrane, as well as activated endogenous Ca^2+^ channels or release of Ca^2+^ from internal stores.

We exposed S-HEK and NS-HEK cells to the same pulse sequence as in ElectroFluor630 experiments (Fig. 2a). We observed Ca^2+^ increase in both S-HEK and NS-HEK already at the lowest E, 126 V/cm (Fig. 5a-c). Ca^2+^ increase appeared to occur slightly more frequently in S-HEK cells than in NS-HEK cells at the lower E, however, the differences were not statistically significant except for 150 V/cm (Fig. 5c). A slow rising of the signal baseline (Fig. 5a,b, see also Supplement 1, Fig. S3) that was observed during the experiment may be caused by a slow Ca^2+^ leak into cells due to experimental conditions (room temperature, low K Tyrode buffer).

We noticed different responses in S-HEK and NS-HEK cells. At the lowest *E*, a small single Ca^2+^ peak occurred immediately after pulse exposure in both S-HEK and NS-HEK cells. At intermediate E, 176 V/cm and 200 V/cm, we unexpectedly observed a complex Ca^2+^ response in S-HEK cells (Fig. 5a,d): after an initial Ca^2+^ peak recovery, a second Ca^2+^ peak occurred that was even higher than the first one. A two-peak Ca^2+^ response was not observed in NS-HEK cells (Fig. 5b,e). Similar to TMV (Fig. 2d), the Ca^2+^ responses vary at lower voltages (Fig. 5d). The larger second peak in S-HEK cells led to a slightly higher maximum of Ca^2+^ response at 150–200 V/cm (S-HEK and NS-HEK cells were significantly different at 176 V/cm), compared to NS-HEK cells (Fig. 5f).

Since the Fura-2 ratio was averaged for the whole image, two peaks may be a result of two events occurring in each cell or one event/peak in two subpopulations of cells at different time points. Therefore, we further analysed 10 individual S-HEK cells in one experiment at 200 V/cm (see Supplement 1, Fig. S4) to evaluate the Ca^2+^ response to the pulse in each cell. The results show that the majority of cells exhibited two Ca^2+^ peaks so the two-peak Ca^2+^ response seem to have occurred on a cellular level. Moreover, when S-HEK cells were exposed to a pulse of 400 V/cm (after the sequence was completed) and observed on a longer time scale (5 min) a similar two-peak Ca^2+^ response was observed (Supplement 1, Fig. S5).

As can be seen from Fig. 5c,d,f, control S-HEK cells exhibited some spontaneous Ca^2+^ transients identified as Ca^2+^ peaks (observed in almost half of experiments). These Ca^2+^ transients are also clearly seen in time lapse images. Such Ca^2+^ transients were not observed in NS-HEK control cells (Fig. 5c,e,f).

### Computational modelling of transmembrane voltage responses

**Figure 6:**
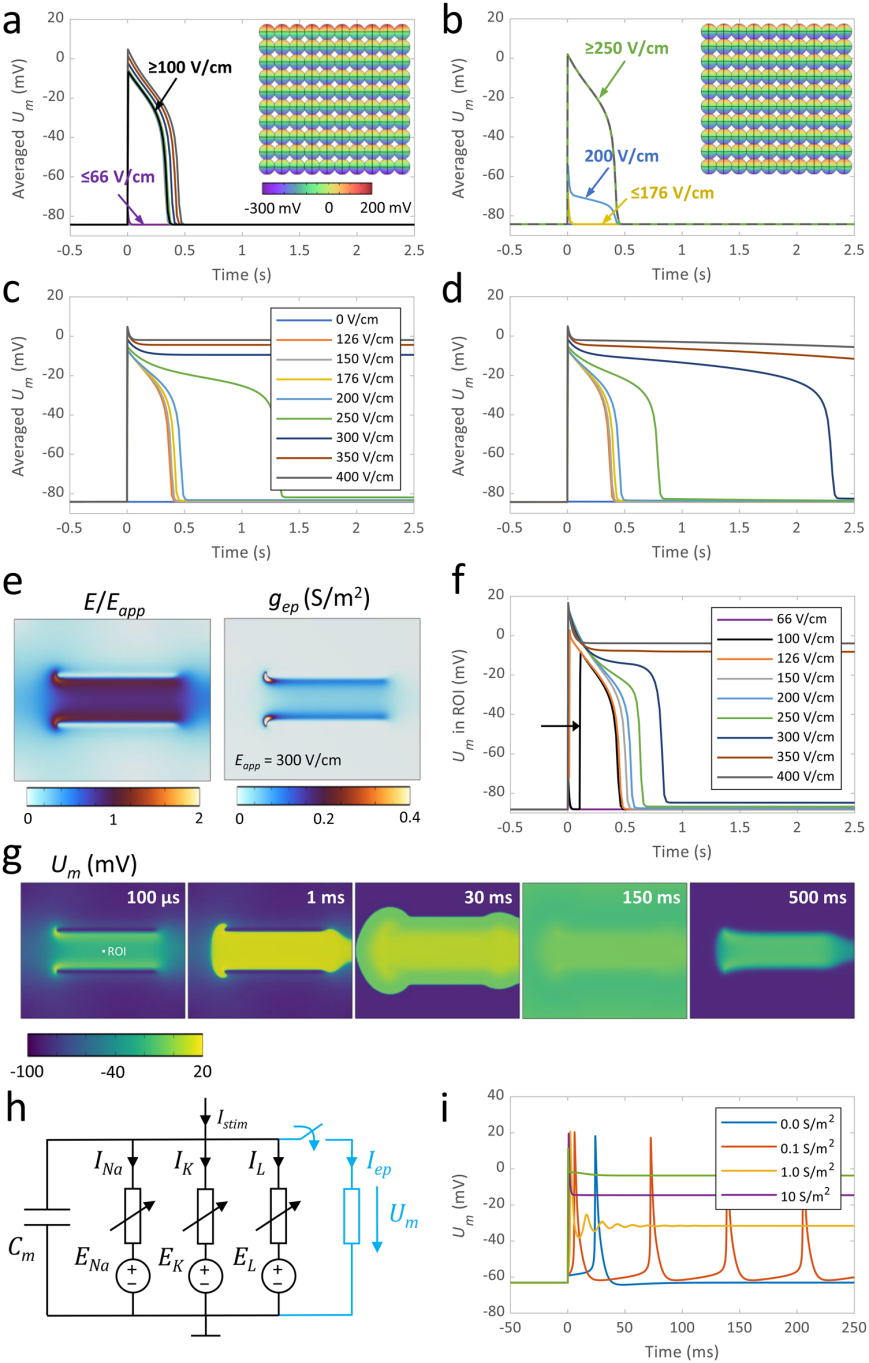
(**a**–**g**) Modelling TMV response in S-HEK cells. (**a**) Response in TMV, denoted by *U*_*m*_, averaged over the membranes of all cells in the cell array model, depending on the applied electric field strength. The inset shows the distribution of the induced *U*_*m*_ at the end of the applied 100 μs pulse. Cells are considered to be connected with gap junctions. (**b**) Same results as in (**a**), but considering that cells are not connected with gap junctions. (**c**) TMV response in the cell array model after including a description for the increase in membrane conductance *g*_*ep*_ due to electroporation. *g*_*ep*_ stays constant until the end of observation time. (**d**) Same results as in (**c**) but considering that *g*_*ep*_ recovers exponentially with characteristic time of 2 s. (**e**) Distribution of the normalized electric field and *g*_*ep*_ in the model of the cell monolayer. *E*_*app*_ is the ratio between the applied voltage and electrode distance. (**f**) TMV response in region of interest (ROI) at the middle between the electrodes computed with the model of the cell monolayer. (**g**) Snapshots showing AP propagation along the monolayer and sustained depolarization between the electrodes when *E*_*app*_ = 300 V/cm. Pulse is applied at time *t* = 0 s. (**h**,**i**) Modelling TMV response in a neuronal cell model. (**h**) Hodgkin-Huxley type equivalent circuit model with added resistor representing the increase in membrane conductance *g*_*ep*_ due to electroporation. (**i**) Computed TMV responses for different values of *g*_*ep*_. *g*_*ep*_ stays constant until the end of observation time.

**Figure 7:**
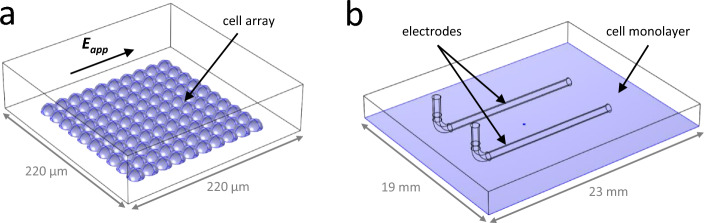
Geometries of the computational models. (**a**) The model of an array of 10 × 10 connected cells (blue) inside a rectangular box representing the extracellular medium. (**b**) The model of a cell monolayer (blue) at the bottom of a well of a Lab-Tek chamber containing two wire electrodes.

To gain a better understanding of the observed TMV responses, we built a 3D finite element model of an idealized array of S-HEK cells (see Fig. 7a in “Materials and methods”), mimicking cells within the field of view imaged in experiments (Fig. 1a,b). We computed the response in TMV to single 100 µs pulses of different amplitudes, as used in the experiments. To facilitate comparison with experiments, we averaged the TMV over the surface area of all cells in the array. We first considered two alternatives. In the first, the cells were electrically connected through gap junctions, which we modelled as an additional ohmic current through the contact areas between cells. In the second, the gap junction current was set to zero. In connected cells, an AP was observed for applied electric field of ≥ 100 V/cm (Fig. 6a), similarly as in experiments (Fig. 2 and Supplement 1, Table S1). In non-connected cells, an AP was observed at applied electric field of ≥ 250 V/cm (Fig. 6b), which is considerably higher than observed experimentally. Spatial profiles of the TMV, that is induced by the end of the 100 µs pulse (insets in Fig. 6a,b), show that the induced TMV is higher by absolute value in connected cells, facing electrodes (top and bottom side of the cell array in the insets). In non-connected cells, the cells at the top and bottom side of the cell array also have a slightly higher induced TMV that can trigger an AP already at an applied electric field of 200 V/cm; however, the AP from these cells cannot propagate to other cells. On the contrary, if cells are electrically connected, the cell array behaves as a syncytium and an AP is generated in all cells in the array practically simultaneously. These results demonstrate that intercellular connectivity through gap junctions has an important influence in the observed S-HEK cell response.

However, in Fig. 6a,b, the shape of the computed TMV response remains the same for all electric fields above the threshold for AP triggering, which does not agree with experimental results. Thus, we hypothesized that the observed change in TMV response with increasing electric field is caused by plasma membrane electroporation. Indeed, the computed induced TMV at the end of a 100 µs, 150 V/cm pulse already exceeded − 300 mV in cells at the anodic side of the cell array. Such high TMV is typically sufficient to induce structural changes in the membrane that increase membrane permeability and electrical conductance. To account for electroporation, we upgraded our model of electrically connected cells with a description relating the increase in membrane conductance *g*_*ep*_ to the induced TMV (see Eq. 10). In the first set of simulations, we considered that *g*_*ep*_ remains constant after the pulse (Fig. 6c), and in the second set of simulations we considered that membrane recovers with time and that *g*_*ep*_ exponentially decays (representing membrane resealing) with an illustrative characteristic time of 2 s (Fig. 6d). The upgraded model showed generation of single APs at the lowest electric field strengths and sustained depolarization at the highest electric field strengths, consistent with experiment. For intermediate electric field strengths, the model showed prolongation of the AP duration (TMV response), which is also in agreement with experimental results (see Fig. 3e).

The cell array model represents a small patch of cells over a length scale of ~ 200 µm. In experiments, cells were grown in monolayer and were exposed to pulses through two electrodes separated by 5 mm, meaning that AP could propagate along the monolayer. We thus built a macroscopic model, representing the entire well of the Lab-Tek chamber with electrodes, with which we simulated AP propagation. This model included a similar description for ionic currents through ion channels, gap junctions, and electroporated membrane as the cell array model. The results show that the electric field distribution in between parallel wire electrodes is not perfectly homogenous, but somewhat higher at the edges along the electrodes (Fig. 6e). Simulation of AP propagation confirmed that AP is first triggered at the electrodes and then propagates along the entire surface of the Lab-Tek chamber (Fig. 6g). In our experiments we always monitored cells that were roughly in the middle between the electrodes; TMV responses computed and plotted for this location (Fig. 6f) are characteristically very similar as obtained with the cell array. In addition, this model shows a delay between the onset of the delivered pulse and detected AP at the lowest applied electric field strength. Note that sustained depolarization in this model occurs at higher electric field than in the cell array model; this is because we derived the expression for the electroporation current based on analytical expression for the induced TMV in a single isolated cell (see “Materials and methods”). Therefore, *g*_*ep*_ for a given electric field in the macroscopic model ends up being lower than in a cell at the edge of the cell array model.

In none of the simulations presented above we observed anything that could explain generation of multiple APs in S-HEK cells. We explored whether an increase in membrane conductance due to electroporation could lead to generation of multiple APs in another excitable cell model. We used a simple Hodgkin-Huxley-type equivalent electric circuit of a neuronal cell and added an additional resistor representing the increase in membrane conductance *g*_*ep*_ due to electroporation. Interestingly, the model showed generation of multiple APs at intermediate *g*_*ep*_, along with sustained depolarization at higher *g*_*ep*_ (Fig. 6h,i). The order of magnitude of *g*_*ep*_, which begins to influence the TMV response (0.1 S/m^2^, see Fig. 6e and i), is comparable to or lower than the maximal conductances of the ion channels in the model (e.g., *g*_*Na*_ = 27.5 S/m^2^, *g*_*K*_ = 0.5 S/m^2^, *g*_*L*_ = 0.05 S/m^2^ in the S-HEK model, see Supplement 2).

## Discussion

### Exciting the S-HEK cells

Experimental studies of effects of high-intensity electric fields on excitable cells such as cardiac cells have remained challenging due to numerous drawbacks of in vitro cell models (primary cardiomyocytes, cardiac cell lines, stem cell-derived cardiomyocytes)^24–26^. Moreover, the variety of voltage-gated and other ion channels that are expressed in different cells^46,47^ makes it challenging to properly understand the consequences of exposing excitable cells to electric pulses. Therefore, a simple cell system with a well-known set of channels obtained by synthetic biology can be a valuable tool to study the mechanisms by which electroporation affects cell excitability.

For this purpose, we used a genetically engineered HEK cell line that expresses a pair of sodium and potassium channels (Na_V_1.5 and K_ir_2.1) that are sufficient to produce APs in otherwise electrically non-excitable HEK cells^30–32^. We electrically stimulated S-HEK cells by delivering a single 100 µs electric pulse. We monitored APs in S-HEK cells with the use of a potentiometric probe ElectroFluor630 and epifluorescence microscopy. S-HEK cells responded to a single electric pulse with single or multiple APs, confirming that these cells are excitable. On the contrary, NS-HEK cells with only Na_V_1.5 and without K_ir_2.1 channels did not respond to electric pulse delivery by triggering APs confirming that they represent a non-excitable version of these cells. S-HEK and NS-HEK cells thus allow us to observe and directly compared the effects of electroporation on TMV changes in excitable and non-excitable counterparts.

### Sustained depolarization and Ca^2+^ increase in S-HEK and NS-HEK cells can be explained by electroporation

At higher electric fields we observed a sustained depolarization in both S-HEK and NS-HEK cells. This sustained depolarization can be explained by an increased membrane conductivity that was predicted also by our computational models. The increased membrane conductivity can be considered a hallmark of electroporation that causes a non-selective ion leak^48^. Electroporation in the narrow sense is widely recognized as a state of increased plasma membrane permeability due to the formation of hydrophilic pores in lipid bilayer, however, the increasing evidence shows that there are also other events that contribute to this non-selective ion leak and/or sustained depolarization, such as electric pulse-triggered chemical changes to the lipids (oxidation) and modulation or damage of ion channels and pumps^21^. It has been shown previously both experimentally^49–55^ and with molecular dynamics simulation^56^ that pulsed electric fields can damage ion channels and pumps and/or modulate their function.

Although we did not detect electroporation by a standard propidium uptake assay, we detected an increase in intracellular Ca^2+^ after pulse delivery. The short depolarization and Ca^2+^ uptake after pulse delivery in NS-HEK cells can point to an existing ion leak^48,57^ that can be a consequence of either permeabilization of the lipid bilayer or ion channel damage. Detection of membrane permeabilization is detection method sensitive (small vs. large dyes)^58^. Electroporation can cause membrane permeability change/openings that allow ions to pass through but that are otherwise too small to pass a much bigger propidium molecule^59–62^. Electric pulse-triggered ion channel damage may also result in pores in protein structure that are smaller than 1.5 nm^56^, thus too small to pass propidium molecule through but enough to pass Ca^2+^ ions^60^.

Our results on non-excitable NS-HEK cells suggest that a small ions leak could be a result of electroporation and is present even at low electric fields (126 V/cm at a single 100 µs electric pulse). Furthermore, our computational models confirm that the TMV response is influenced by electoporation already at the lowest electric fields used in experiments, which were close to the stimulation threshold. Conventionally, in vitro electrostimulation is done with millisecond pulses^63^. Our results suggest that when applying 100 µs pulses, as used conventionally in electroporation applications, electroporation may exist already at electric fields close to the stimulation thresholds. However, such electroporation is short-lived (see the % of TMV responses and returns to the baseline in NS-HEK cells) and of low extent (non-detectable by propidium uptake). These results corroborate the findings of other groups showing that when 100 µs electric pulses are used, the stimulation is much lower than the propidium-detectable electroporation threshold (one order of magnitude)^64^ or irreversible electroporation (almost three orders of magnitude)^12,65^.

Such weak electroporation (undetectable by standard electroporation assays^58^) can, at least in part, play a role in the delayed triggering of APs at low electric field, as seen in our experimental results: weak electroporation may cause a slow depolarization that leads eventually to AP initiation. It was suggested that in stimulation with electric pulses of nanosecond duration (nsEP), the APs result not directly from the nsEP pulses (by activating ion channels) but from ions that leak across the membrane permeabilized by nsEP^57,66^. Accordingly, a similar, amplitude-dependent AP delay was observed when using nsEP pulses^67^. Additional explanation for the delay in triggering APs at lower electric field comes from our computational modelling: immediately next to the electrodes, the electric field is substantially higher than in the middle between the electrodes and cells in the area close to the electrodes are excited. The cells in the monolayer are interconnected so APs propagate from the electrodes to the middle position between the electrodes where image acquisition is done. With the approximate distance of 2.5 mm and a rough estimation of AP propagation velocity of 34 mm/s (estimated on images with much smaller magnification—with 5× objective, see Supplement 1, Fig. S6), the propagation of AP from the electrodes to the middle position can result in the delay in the range of several tens of ms, accounting for a considerable fraction of the experimentally determined delay (see Fig. 3f). Note that we could not observe AP propagation in the images captured with 40× objective (used for monitoring TMV responses), since the side length of our square images is roughly 0.33 mm; the AP would travel this length in roughly 10 ms, which is shorter than our image acquisition (one image each 36 ms).

### Generation of multiple action potentials in S-HEK cells

A small nonselective leakage of ions could also explain repetitive firing (multiple APs) observed in our experiments. Namely, it is known that a repetitive response in neurons occurs due to a steady, persistent depolarizing current, mostly sodium^68,69^. Such repetitive firing was also observed in our electric circuit model of neuronal AP upon increasing the membrane conductance. However, we were unable to observe repetitive firing in our models of S-HEK cells. The discrepancy between the models and experiment can be due to at least two reasons. HEK cells are known to express multiple endogenous voltage-gated ion channels, including sodium voltage-gated channels^70^, potassium voltage-gated channels^71,72^, chloride channels^73^, and various types of calcium channels (discussed in further detail below). While HEK cells are generally considered as suitable host for electrophysiological studies of transiently expressed ion channels, the endogenous currents can be large enough to influence the electrophysiological response in stably transfected cells^72^. Thus, the repetitive firing might be related to currents not implemented and accounted for in our models. Consistent with previous publications^31^, we observed that S-HEK cells in dense monolayers can spontaneously spike periodically, whereby such periodic firing cannot be explained by the mathematical description of Na_V_1.5 and K_ir_2.1 channels used in the model^40^. Furthermore, our models do not consider changes in intracellular ionic concentrations (due to currents through ion channels or electroporation), which plays an important role in the electrophysiological response of the cell.

### A complex calcium response to electric pulses in excitable S-HEK cells

In our experiments, intracellular Ca^2+^ increased in response to pulse delivery and the increase became greater with increasing electric field of the delivered pulses. Interestingly, we also observed a complex Ca^2+^ response in excitable S-HEK cells but not in non-excitable NS-HEK cells. After the initial peak in Ca^2+^ increase (presumably resulting from electroporation, opening of voltage-gated calcium channels, or leak through some other channels), a second, usually larger peak was observed. The second peak in S-HEK cells can be caused by opening of endogenous Ca^2+^ channels in the plasma membrane, Ca^2+^ passing through other channels such as Transient Receptor Potential (TRP) channels^74^, or more complex Ca^2+^-related pathways such as calcium-induced calcium release (CICR)^75^, store-operated calcium entry^76^ or intercellular calcium waves^77^. The data from Human Protein Atlas for HEK293 cells (https://www.proteinatlas.org/search/HEK293) as well as from the literature^31,78–80^ revealed that these cells express channels and receptors involved in all these pathways that may potentially cause the second Ca^2+^ peak in S-HEK cells. To identify the mechanism responsible for the second calcium peak, other methods would need to be applied (channel blockers, depletion of internal calcium stores, detailed spatial observation of Ca^2+^ increase on a cellular and cell monolayer level, gene expression).

Nevertheless, S-HEK and NS-HEK cells differ in such a way that a second calcium peak is seen only in S-HEK cells. The expression of K_ir_2.1 channels only in S-HEK may have an unknown additional effect on other ion channels and proteins that may influence the Ca^2+^ response^81,82^. Moreover, related to the presence or absence of K_ir_2.1 channels, S-HEK and NS-HEK cells also differ in the resting TMV: excitable S-HEK cells and non-excitable NS-HEK have resting TMV around -70 mV and -20 mV, respectively^31,45^. Some of the difference in responses between S-HEK and NS-HEK cells could thus also be related to their difference in the resting TMV.

With higher electric fields (> 300 V/cm), we detected a sustained increase (more than a minute) in intracellular Ca^2+^ concentration, similar to other authors who attribute such observations to electroporation^16,62,67,83–86^. Increased intracellular Ca^2+^ levels activates a number of cell responses, namely, contraction, secretion of neurotransmitters, channel gating, activation of metabolic and signalling enzymes and gene expression^68^. Importantly, Ca^2+^ currents also shape APs^68^. A massive Ca^2+^ influx in our experiments can have profound consequences such as protein aggregation, proteolysis and mitochondrial dysfunction, which can lead to asynchronous firing or a delayed cell death^16,62,87,88^.

## Conclusions

By comparing the responses of S-HEK cells and their non-excitable counterparts (NS-HEK cells) to single 100 µs pulses we found that AP generation in S-HEK cells becomes affected by electroporation already at low electric fields that are close to the electrostimulation threshold. The effects of electroporation were manifested in generation of multiple APs, AP prolongation and, at higher electric fields, sustained membrane depolarization. All these effects were observed at very mild conditions in the absence of detectable propidium uptake, which is a classical indicator of electroporation, but were associated with significant increase in intracellular calcium. Our computational modelling demonstrated that the observed effects can be largely explained by a small increase in membrane ionic conductance due to electroporation, which is comparable to the maximum conductance of ion channels expressed in the membrane. Our study thus demonstrates that changes in AP generation are a sensitive indicator of very mild membrane perturbation and cannot be used as an indicator of irreversible membrane damage.

However, our computational models were unable to explain spontaneous generation of multiple APs and complex intracellular Ca^2+^ dynamics observed in S-HEK cells at low and intermediate electric fields. One of the drawbacks of HEK cells is that they express various endogenous ion channels, which could contribute to the observed response. Additionally, the electric pulses could alter the function of Na_V_1.5 and K_ir_2.1 ion channels. Nevertheless, S-HEK cells offer several tools that can help develop more accurate models and decipher complex responses of excitable cells to electroporation in future studies. The expression of endogenous channels in HEK cells has been relatively well characterized, thus the contribution of these channels to S-HEK cell response can be assessed with corresponding ion channel modulators. Furthermore, studying the effects of intense (supraphysiological) electric pulses on Na_V_1.5 and K_ir_2.1 channels is possible with molecular dynamics simulations since their structure was identified^34,35^.

It should be stressed that S-HEK cells mimic native excitable cells only in terms of their ability to generate and propagate APs, but not in other characteristics such as excitation–contraction coupling in myocytes. While on the one hand this is a limitation, on the other hand this acts as an advantage, as we can use S-HEK cells to study the effects of electroporation solely on cell excitability without other confounding factors. Overall, we find S-HEK cells to be a valuable in vitro experimental system that can be efficiently coupled with theoretical modelling to develop a mechanistic understanding of electroporation of excitable cells. As such we will be able to better understand the mechanisms underlying different responses and outcomes in rapidly emerging electroporation-based therapies in the heart (PFA ablation for treatment of heart arrhythmias^89^, gene therapies for heart regeneration^90^), brain (tumour ablation^91^, treating epilepsy^43^), and skeletal muscles (gene therapies, DNA vaccinations^92^). This new knowledge will enable better prediction of treatment outcomes and optimization of protocols for these treatments.

## Materials and methods

### Cells

The genetically engineered HEK cell line was developed and generously provided by the group of Adam E. Cohen at Harvard University^31,32^. The cell line was derived from HEK-293T cell line (ATCC CRL-3216) and genetically modified for constitutive expression Na_V_1.5 channels and with a tet-on system of doxycycline-induced expression of K_ir_2.1 channels. Cells with both Na_V_1.5 and K_ir_2.1 channels are able to generate APs (“spikes”), therefore, they served as a model of excitable cells (spiking S-HEK cells). Cells with only Na_V_1.5 channels served as complementary non-excitable cells (non-spiking NS-HEK cells). These tet-on spiking HEK cells have since become available from ATCC (cat. no. CRL-3479). The cell line was propagated from week to week as NS-HEK cells, in Dulbecco’s Modified Eagle’s Medium high glucose growth medium (D5671, Sigma-Aldrich/Merck KGaA, Darmstadt, Germany), supplemented with 10% fetal bovine serum (F2442, Sigma-Aldrich/Merck), 2 mM glutamine (G7513, Sigma-Aldrich/Merck), penicillin–streptomycin (P07681, Sigma-Aldrich/Merck; penicillin 100 U/ml, streptomycin 100 µg/ml), 2 µg/ml puromycin (Thermo Fisher Scientific Inc., Waltham, Massachusetts, U.S.), 5 µg/ml blasticidin (Thermo Fisher Scientific), and 200 µg/ml geneticin (Thermo Fisher Scientific).

Cells were seeded two or three days prior to experiments (2.5 × 10^5^ or 10^5^ cells seeded per well, respectively) to poly-d-lysine-coated 2-well Nunc Lab-Tek II Chambered #1.5 German Coverglass System (155379, Thermo Fisher Scientific) for ElectroFluor630 and Fura-2 experiments, or 2-well Nunc Lab-Tek II chambers with Permanox plastic bottom (177429, Thermo Fisher Scientific) for propidium experiments. For generation of S-HEK cells, 4 µg/ml doxycycline (D9891, Sigma-Aldrich/Merck) was added to cells when seeded for a doxycycline-induced expression of K_ir_2.1 channels. A successful expression of K_ir_2.1 channels was confirmed by verification of appearance of Cyan Fluorescent Protein that is co-expressed with K_ir_2.1 channels and doxycycline dose-dependent (Supplement 1, Fig. S7). NS-HEK cells were seeded in chambers without doxycycline added and with 10% fewer cells (they grow faster than S-HEK). Only passages lower than 14 were used in the experiments.

### Monitoring changes in transmembrane voltage

For monitoring changes in transmembrane voltage (TMV), including APs, ElectroFluor630 (Di-4-ANEQ(F)PTEA) from Potentiometric Probes, Farmington, CT, USA was used^93,94^. The dye was chosen to avoid potential crosstalk with Cyan Fluorescent Protein, that is co-expressed with K_ir_2.1 channels in S-HEK cells and enables visual confirmation of successful K_ir_2.1 expression. The intensity of the potentiometric dye is linearly proportional to the TMV at least in the range between − 80 to + 20 mV^94^, however, the impact of membrane electroporation on the brightness of the dye was not yet studied. Cells were labelled with 12 µM ElectroFluor630 dye in culture medium for 20 min in a refrigerator at ~ 4 °C, 3× washed with Tyrode buffer (abbreviated here as TB; 2 mM KCl, 125 mM NaCl, 2 mM CaCl_2_, 1 mM MgCl_2_, 10 mM HEPES, 30 mM glucose, pH 7.3). At the end, a low K^+^ TB (0.5 mM KCl, 126.5 mM NaCl, other components the same as in TB) was added to cells for more robust triggering of APs. All experiments were performed at room temperature.

Each sample of cells was exposed to a sequence of eight 100 µs pulses of increasing voltage of 63, 75, 88, 100, 125, 150, 175, and 200 V, delivered 2 min apart (for better clarity see Fig. 2a) to minimize the possible cumulative effects of consecutive pulses^83^ while keeping experiments short enough not to deteriorate cells. The use of fresh cells for each pulse amplitude (which would be ideal to eliminate the possibility of cumulative effects) would be prohibitively time- and cost- consuming and therefore not practical. Pulses were delivered with a laboratory prototype pulse generator^95,96^ connected to two parallel Pt/Ir wire electrodes, with 0.8 mm diameter and 5 mm distance between inner edges, placed at the bottom of the Lab-Tek chamber. Voltage and current were measured using a WavePro 7300A oscilloscope with a differential voltage probe ADP305 and a current probe AP015, all from Teledyne LeCroy, Chestnut Ridge, NY, USA. The electric field to which the cells were exposed was estimated as the applied voltage-to-electrode-distance ratio (*E* ≈ 126, 150, 176, 200, 250, 300, 350, and 400 V/cm). Since we observed APs in S-HEK cells already at the lowest *E*, we also exposed cells to a sequence of pulses with 20, 33, and 50 V (*E* ≈ 40, 66, and 100 V/cm) in separate experiments.

The electric current flowing through the sample when delivering a 100 µs pulse of the highest amplitude tested in experiments (200 V) was 1.74 A, which corresponds to the power of 348 W.

We also estimated the temperature rise in the medium between the electrodes according to a “worst-case” scenario, where the energy of a pulse is transformed into heat in one moment without any heat dissipation^97^.

1 $$\Delta T=\frac{\Delta W}{{c}_{m}m}=\frac{{E}^{2}\sigma {t}_{pulse}}{{c}_{m}\rho }=\frac{{\left(400 \;{\text{V}}/{\text{cm}}\right)}^{2}\cdot 1.432 \;\mathrm{ S}/{\text{m}}\cdot 100\; { \upmu {\text{s}}}}{4200\; {\text{J}}/\left({\text{kg}}\cdot {\text{K}}\right)\cdot 1000 \; \mathrm{ kg}/{{\text{m}}}^{3}}=0.05\; {\text{K}}$$

For the highest *E* tested in experiments (400 V/cm), the calculated temperature rise is 0.05 K, which has a negligible effect on cell functions. Moreover, since the delay between the applied pulses was 2 min, the heat generated by each pulse had enough time to dissipate into the environment.

Cells were observed under a Thunder Imager Live Cell system for fluorescence microscopy with DMi8 inverted fluorescent microscope, deep-cooled 4.2 MP sCMOS Leica DFC9000 Gt fast camera, a system of 8 LED diodes (LED8), and a UV light source (CoolLED pE340fura) (all from Leica Microsystems GmbH, Wetzlar, Germany). For monitoring APs and changes in TMV, cells were exposed to electric pulses under the microscope in the dark. During the delivery of each pulse, images were acquired with the LAS X software (Leica Microsystems) in time-lapse acquisition mode (2.8 s total duration of image acquisition, 80 images, one image every 36 ms). Pulse delivery was synchronized with image acquisition: after the 10th image (at 324 ms) a TTL signal from the microscope system triggered the pulse generator. In each experiment, a time-lapse without any pulse exposure was acquired at the beginning to serve as a control. Cells were illuminated with excitation wavelength of 635 nm at 50% LED power, exposure time of 10 ms, and 4 × 4 binning; the emission was detected at 700 nm wavelength using an appropriate filter set (DFT51010), and observed with a 40× objective. Image analysis was done with a custom Matlab algorithm to extract relevant parameters (see below).

### Analysis of changes in transmembrane voltage

The ElectroFluor630 fluorescence signal was analysed using Matlab (Mathworks, Natick, MA, USA). A custom Matlab application was created to automate the image processing and extract the relevant parameters of the fluorescent responses. The app was designed to first threshold the images (with a low threshold and a high threshold). This allowed the user to remove any obvious debris and focus the analysis on the well-defined membrane regions to improve the signal-to-noise ratio. The application then performed automatic fluorescence signal analysis, which consisted of removing the fluorescence fading, detecting the peak intensity changes, and extracting the time parameters of the signal (Supplement 1, Fig. S8).

The average fluorescence intensity of all thresholded pixels for each delivered consecutive pulse was calculated to obtain a time-dependent signal of fluorescence *F*. Because each experimental run consisted of a control image sequence in which no pulse was delivered, the control sequence was used to fit a linear function with slope *k* describing the fading of the dye (Supplement 1, Fig. S9). This slope *k* was then used to correct for the fading in the signal *F*. We first determined the linear function:

2 $${F}_{0}=k\cdot t+{F}_{intercept}$$

where *F*_*intercept*_ was set to the mean value of the 10 measured fluorescence values before the pulse for each segment of *F* corresponding to the response obtained for a given pulse in the pulse sequence. We then determined the relative change in fluorescence as:

3 $$R=1-\frac{F}{{F}_{0}}$$

since the relative decrease in ElectroFluor630 fluorescence is linearly proportional to the increase in TMV^94^. This fluorescence response signal *R* (see Fig. 1c) was then processed to extract the following parameters: number of peaks, average peak-to-peak time (when more than one peak was visible), maximum response, time to first peak, and time from peak to 25%, 50%, and 90% recovery. The maximum response was simply the maximum value of *R*. Time to first peak was determined by subtracting the time of pulse delivery from the time of the first peak. The time to x% recovery was determined by finding the first time point at which the signal decreased by 25%, 50%, or 90% from the value of the maximum response.

The location and number of peaks were determined either by using Matlab's built-in peak finder function when APs were present, or by finding the first point above a minimum threshold (relative change of 0.008) when only depolarization was present. All automatic peak locations were checked visually by the investigators. In some cases, when the algorithm did not detect an obvious peak, or when the algorithm detected spurious noise signals as false positives, the user had the option to manually select the corresponding point in the fluorescence signal as a peak or to remove the detected false peak.

Based on the obtained results, we classified the responses into 4 main groups: no response (no peaks detected), single APs, multiple APs (multiple peaks detected), and sustained depolarization. To discriminate between single APs and sustained depolarization, which were both characterized by a single peak, we first determined typical characteristics of APs that appear after stimulation with low-intensity 100 µs pulses in our experiments. We collected all responses of S-HEK cells at the lowest electric field that induced a response and defined a single AP as a response that has a peak amplitude > 0.02, time to 50% recovery < 700 ms, and ratio between time to 90% and 50% recovery < 2. All other single peak responses were classified as sustained depolarization.

### Monitoring electroporation with propidium uptake

Propidium uptake was used as a standard means for electroporation detection. For detecting propidium uptake, cells were first labelled with 4 µM Hoechst 33342 dye (62,249, Thermo Fisher Scientific) that labels all cell nuclei (7 min at 37 °C, 3 × washed with TB). Labelling with Hoechst facilitated image analysis. The low K^+^ TB with 50 µM propidium iodide (P1304MP, Thermo Fisher Scientific) was then added to cells for experiments. All experiments were performed at room temperature.

Cells with added propidium were exposed to the same sequence of eight pulses delivered two min apart as in ElectroFluor630 experiments, see above. We also tested propidium uptake after exposing the cells to a train of 8 × 100 µs pulses of 400 V/cm or 1000 V/cm, 1 Hz. 8 × 100 µs, 1000 V/cm, 1 Hz pulses were chosen as a positive control as pulses that are typically used in electrochemotherapy and in vitro electroporation^98^. 8 × 100 µs, 400 V/cm, 1 Hz pulses were chosen as eight pulses of the highest electric field from ElectroFluor630 experiments. Pulses were delivered in the laminar flow hood with an Electro cell B10 electroporator (BetaTech, Saint-Orens-de-Gameville, France) using the same pulse waveforms, electrodes, oscilloscope and voltage and current probes as in ElectroFluor630. 5 min after pulse delivery, cells were washed once with TB and transferred under the microscope. Two controls with no pulses delivered were tested for propidium uptake for each pulsing protocol: after 20 min of incubation with propidium (the same time frame as with pulse sequence from ElectroFluor630 experiments) and after 5 min (for trains of 8 × 100 µs pulses of 400 V/cm or 1000 V/cm, 1 Hz).

For monitoring electroporation with propidium uptake, cells were observed under the Thunder fluorescence microscope with excitation wavelength of 555 nm at 10% LED power, exposure time of 100 ms, and no binning; the emission was detected at 600 nm wavelength using an appropriate filter set (DFT51010), and observed with a 40× objective. Hoechst 33342 images were acquired with excitation wavelength of 390 nm at 30% LED power, exposure time of 100 ms, and no binning; the emission was detected at 440 nm wavelength using an appropriate filter set (DFT51010). At each experiment, images of 2–3 fields of view were acquired.

Uptake of propidium was evaluated with an open-source image-processing program ImageJ (National Institutes of Health, Bethesda, MD)^99^. Hoechst images were thresholded to obtain a mask around the nuclei. The mask of nuclei was then used to set ROI of propidium images of the same experiment and an average propidium fluorescence intensity in the nuclear area was determined. Average background was subtracted from the results. The described analytical approach allowed us to automatically quantify the propidium fluorescence within cells, even in samples that were not detectably stained with propidium.

### Monitoring electroporation with calcium uptake

Intracellular Ca^2+^ was monitored using the fluorescent Ca^2+^ indicator Fura-2 acetoxymethyl (AM) ester (F1221, ThermoFisher Scientific). Changes in intracellular Ca^2+^ concentration influence the fluorescence spectrum of Fura-2 which can be detected in ratiometric measurements^58,86,100^.

Cells in Lab-Tek chambers were stained with 2 µM Fura-2 AM in culture medium at 37 °C for 30 min, 3× washed with TB and transferred to low K^+^ TB which contains 2 mM CaCl_2_ (see chapter Monitoring Changes in TMV). Cells were then exposed to the same pulse sequence as in ElectroFluor630 experiments. Pulses were delivered by the Electro cell B10 electroporator (BetaTech) using the same electrodes, oscilloscope and voltage and current probes as in ElectroFluor630 experiments. At each pulse, image acquisition lasted for 40 s. Additionally, at the end of an experiment (5 min after the sequence was completed), cells were exposed to a pulse of 400 V/cm and observed on a longer time scale (5 min) to detect the dynamics and the recovery of the intracellular Ca^2+^ level.

Cells were observed under an inverted epifluorescent microscope (Zeiss Axiovert 200, Zeiss, Oberkochen, Germany) using a 63× objective and Prime sCMOS camera (Photometrics, Tucson, AZ, USA). Fura-2 was used in ratiometric measurements. Cells were illuminated with Xe light source and a monochromator (VisiChrome Polychromator, Visitron, Puchheim, Germany) at two excitation wavelengths (340 nm and 380 nm) and an exposure time of 200 ms for both wavelengths. The emission was detected at 510 nm wavelength using an appropriate filter set (Chroma 71500, Chroma Technology Corporation, Bellows Falls, VT, USA). Images were acquired with the software VisiView (Visitron) in time-lapse acquisition mode (39 s total duration of image acquisition, 40 images, one image every 1 s). Pulses were delivered manually (with no trigger) at the 5th image (at around 5 s). Additionally, 5 min after the 126–400 V/cm pulse sequence was completed, we delivered an additional 400 V/cm pulse and monitored the Ca^2+^ level recovery on a longer time scale (almost 5 min). In this recovery test, the imaging parameters were: 290 s total duration of image acquisition, 30 images, one image every 10 s, where a pulse was delivered at the 5th second of image acquisition.

In the VisiView software, background was subtracted from each image and ratio image Fura-2 340/380 was calculated in each pixel. A higher intracellular Ca^2+^ concentration results in a higher Fura-2 340/380 ratio. The average ratio of the whole image was determined using ImageJ to obtain the Fura-2 ratio *R*_340/380_(*t*) as a function of time for each applied pulse. The curves *R*_340/380_(*t*) were then further analysed by a custom Matlab algorithm to extract relevant parameters. The beginning of each *R*_340/380_(*t*) curve was first shifted to zero, *R*_340/380_(*t* = 0 s) = 0, and then the location of peaks in each curve was determined using Matlab's built-in peak finder function. If a peak was not found with the peak finder function, then the algorithm additionally checked, if there was a zero crossing in the time derivative of *R*_340/380_(*t*). If yes, then a peak was assigned at the time, when the curve reached 95% of the value at the derivative zero crossing. This additional step facilitated automatic detection of peaks in curves that resembled a sigmoidal function. If a peak was not found in the second step, then the curve was assigned as having no peaks. For each curve, we determined the number of peaks and the peak amplitude.

### Statistical analysis

Statistical analysis was performed using Excel (Microsoft, Redmond, WA, USA) and SigmaPlot 11.0 (Systat Software, Chicago, IL, USA). The results in figures and the text are expressed as means ± SD or medians with bars Q1 and Q3. Normality of the data distribution was tested with the Shapiro–Wilk test and since the normality assumption was not fulfilled in majority of cases, the nonparametric tests were used to assess the statistical significance of the differences. Significant differences (p < 0.05) in responses (TMV, Ca^2+^) and recovery of the TMV were determined by Fisher’s Exact test. Significant differences (p < 0.05) in maximal responses (TMV, Ca^2+^), and time to first peak were determined by Mann–Whitney Rank Sum Test. Significant differences (p < 0.05) in propidium uptake were determined by One-way ANOVA on Ranks.

### Computational modelling

Finite element modelling was carried out in Comsol Multiphysics 6.0 (Comsol Inc., Stockholm, Sweden), following our previous experience^101–103^. We developed two main models, one at the microscopic level composed of individual cells and one at the macroscopic level of the entire cell monolayer.

The first model represented an array of 10 × 10 connected S-HEK cells in a rectangular extracellular domain (Fig. 7a). The cells were modelled as hemispheres with radius *R*_*cell*_ = 9 µm, with their centre-to-centre distance of 1.9*R*_*cell*_. Hence, the cells were in contact and each cell shared approximately 10% of its membrane surface area with neighbouring cells. The electric potential distribution *V* in the intracellular and extracellular space was described as:

4 $$\nabla \cdot \left[\left({\sigma }_{i,e}+{\varepsilon }_{i,e}\frac{\partial }{\partial t}\right)\nabla {V}_{i,e}\right]=0$$

where *σ*_*i,e*_ and *ε*_*i,e*_ denote, respectively, the conductivity and the dielectric permittivity of the intracellular (subscript *i*) or extracellular (subscript *e*) medium. Two opposite sides of the rectangular domain were modelled as electrodes by assigning them an electric potential. The positive electrode (anode) was excited by a rectangular 100 µs long pulse of chosen amplitude, while the negative electrode (cathode) was kept at 0 V. Other sides of the box were modelled as electrically insulating. The plasma membrane was modelled as a boundary condition, which describes the continuity of the normal component of the electric current density **J**_***m***_ across the membrane:

5 $$\mathbf{n}\cdot {\mathbf{J}}_{{\varvec{m}}}={C}_{m}\frac{\partial {U}_{m}}{\partial t}+{J}_{Na}+{J}_{K}+{J}_{L}+{g}_{ep}{U}_{m}$$

where **n** denotes the unit vector normal to the membrane surface. The TMV, denoted in equations by *U*_*m*_, was determined as the difference between the electric potentials at the intracellular (*V*_*i*_) and extracellular (*V*_*e*_) side of the membrane, *U*_*m*_ = *V*_*i*_ – *V*_*e*_. The electric current densities *J*_*Na*_, *J*_*K*_, *J*_*L*_ represented, respectively, the currents through Na_V_1.5, K_ir_2.1, and leak channels and were described using Hodgkin-Huxley-type equations, following previous studies in S-HEK cells^38–40^:

6 $${J}_{Na}={g}_{Na}{m}^{3}hj\left({U}_{m}-{E}_{Na}\right)$$

7 $${J}_{K}={g}_{K}{n}_{\infty }\left({U}_{m}-{E}_{K}\right)$$

8 $${J}_{L}= {g}_{L}\left({U}_{m}-{E}_{L}\right)$$

where *g*_*X*_ (*X* = Na, K, or L) is the maximal conductance of the given type of ion channel and *E*_*X*_ is the reversal potential for the given type of ions. The gating variables *m*, *h,* and *j* for Na_V_1.5 channels were determined by solving differential equations of the form:

9 $$\frac{dy}{dt}=\frac{\left({y}_{\infty }-y\right)}{{\tau }_{y}}$$

where *y*_∞_ and *τ*_*y*_ (*y* = *m*, *h*, or *j*) are functions of *U*_*m*_ and describe the transitions between the separate states of the channel. The current through K_ir_2.1 channels was described with the characteristic voltage-dependent function *n*_∞_. The full expressions for *y*_∞_, *τ*_*y*_, and *n*_∞_ are given in the Supplement 2, Section S1.1. The last term in Eq. (5) corresponds to the nonselective current due to membrane electroporation, with *g*_*ep*_ being the associated increase in membrane conductance. To describe *g*_*ep*_, we adapted an empirical expression that was developed in a previous study^104^:

10 $${g}_{ep}=\left\{\begin{array}{ll}\alpha \left({e}^{\beta \left|{U}_{m}\right|}-1\right),& \quad t\le {t}_{pulse}\\ {g}_{ep}\left(t={t}_{pulse}\right){e}^{-\frac{t}{{\tau }_{r}}},& \quad t\ge {t}_{pulse}\end{array}\right.$$

where *α* and *β* are parameters that were fitted to experimental data. We considered that during the pulse *g*_*ep*_ exponentially increases with *U*_*m*_, whereas after the pulse it recovers exponentially with characteristic time *τ*_*r*_. We performed simulations for two conditions, one in which *τ*_*r*_ is much longer than the observation time (*τ*_*r*_ >  > 3 s), and one in which *τ*_*r*_ is shorter than the observation time. In the contact area between cells the electric current density was defined as:

11 $$\mathbf{n}\cdot {\mathbf{J}}_{{\varvec{m}}}=\frac{{C}_{m}}{2}\frac{\partial ({V}_{1}-{V}_{2})}{\partial t}+{g}_{Cxn}({V}_{1}-{V}_{2})$$

where *V*_*1*_ and *V*_*2*_ denote the electric potentials at the two interfaces of the contact area, and *g*_*Cxn*_ is the gap junctions’ conductance, assumed to be time- and voltage-independent^38–40^. We note that assuming a constant gap junction’s conductance is a simplification as gap junctions can exhibit voltage-dependent conductance^105^ which can moreover be affected by intense electric field^106^.

The second model represented the entire cell monolayer within a well of a Lab-Tek chamber containing wire electrodes (Fig. 7b). In simulations with this model, we first computed the local electric field distribution *E* inside the well by solving the steady-state version of Eq. (4) for the extracellular domain. We then used this solution as input to simulations of AP generation and propagation along the monolayer. For AP simulations, we solved the equation^39^:

12 $${C}_{m}\frac{d{U}_{m}}{dt}={G}_{Cxn}{\nabla }^{2}{U}_{m}-{J}_{Na}-{J}_{K}- {J}_{L}-{G}_{ep}{U}_{m}-{J}_{stim}$$

The gap junction conductance at the monolayer level *G*_*Cxn*_ was defined as^39^:

13 $${G}_{Cxn}={g}_{Cxn}{\left(2{R}_{cell}\right)}^{2}$$

where *R*_*cell*_ is the cell radius. The equations describing *J*_*Na*_, *J*_*K*_, *J*_*L*_ were identical as in Eqs. (6–8). To enable simulations that mimic our experimental conditions, we needed to derive an expression that connects the local electric field *E* with the stimulus current *J*_*stim*_. We derived the equation:

14 $${J}_{stim}=-\frac{3}{8}{R}_{cell}E\left({g}_{L}\left(1-{e}^{-\frac{t}{{\tau }_{chg}}}\right)+\frac{{C}_{m}}{{\tau }_{chg}} {e}^{-\frac{t}{{\tau }_{chg}}}\right)\left(h\left(t\right)-h\left(t-{t}_{pulse}\right)\right)$$

The functions *h*(*t*) are smoothed Heaviside functions and were used to describe the applied 100 µs pulse, whereas *τ*_*chg*_ is the characteristic membrane charging time. In addition, we needed to derive an expression that connects the local electric field *E* with the increase in membrane conductance due to electroporation at the monolayer level *G*_*ep*_, considering Eq. (10). We derived the equation:

15 $${G}_{ep}=\alpha \left(\frac{{\text{exp}}\left(1.5{R}_{cell}E\beta \right)-1}{1.5{R}_{cell}E\beta }-1\right)h\left(t\right)$$

The derivation of Eqs. (14) and (15) was based on the analytical expression for the induced TMV in an isolated spherical cell, since it provided an elegant solution. We note that this analytical solution somewhat deviates from the induced TMV in connected cells, but nevertheless the derived Eqs. (14) and (15) were sufficient to characteristically reproduce the responses observed experimentally.

Supplement 2 provides further information on the model design, the rationale for Eq. (10), detailed derivation of Eqs. (14) and (15), the values of all model parameters, and the technical aspects of the model implementation in Comsol Multiphysics.

### Supplementary Information

Supplementary Information 1.

Supplementary Information 2.

## Data Availability

The data presented in this study is available on request from the corresponding author.
