# Supplementary material for: Genetically engineered HEK cells as a valuable tool for studying electroporation in excitable cells
Source: Sci Rep. 2024 Jan 6;14:720. doi: 10.1038/s41598-023-51073-5 (PMC10771480; doi:10.1038/s41598-023-51073-5)
Supplement: Supplementary file 1 — Supplementary Information 1. [file 41598_2023_51073_MOESM1_ESM.pdf]

Genetically engineered HEK cells as a valuable tool for studying electroporation in excitable cells

Tina Batista Napotnik, Bor Kos, Tomaž Jarm, Damijan Miklavčič, Rodney P. O'Connor, Lea Rems

Supplement 1

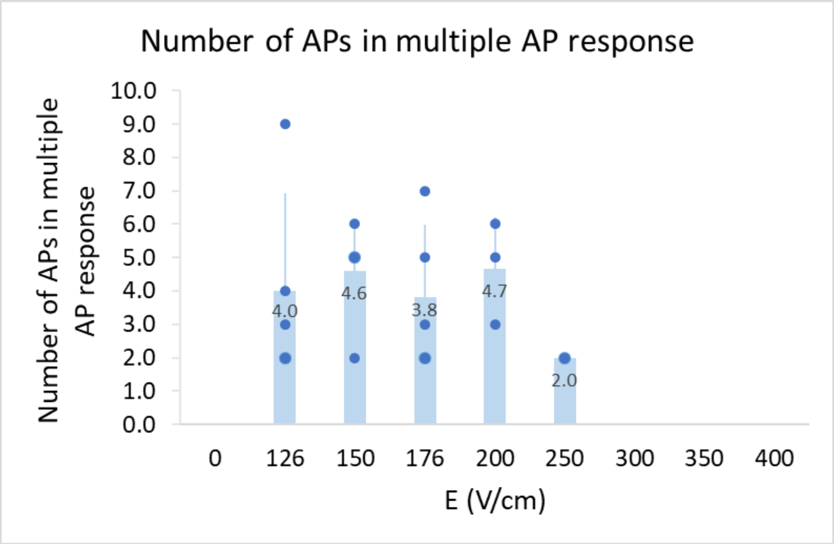

Figure S1: Number of action potentials (APs) in multiple AP responses in excitable S-HEK cells after being exposed to a single 100 µs electric pulses of different electric field. The average number, with data points of APs ±SD in multiple AP responses, are shown.

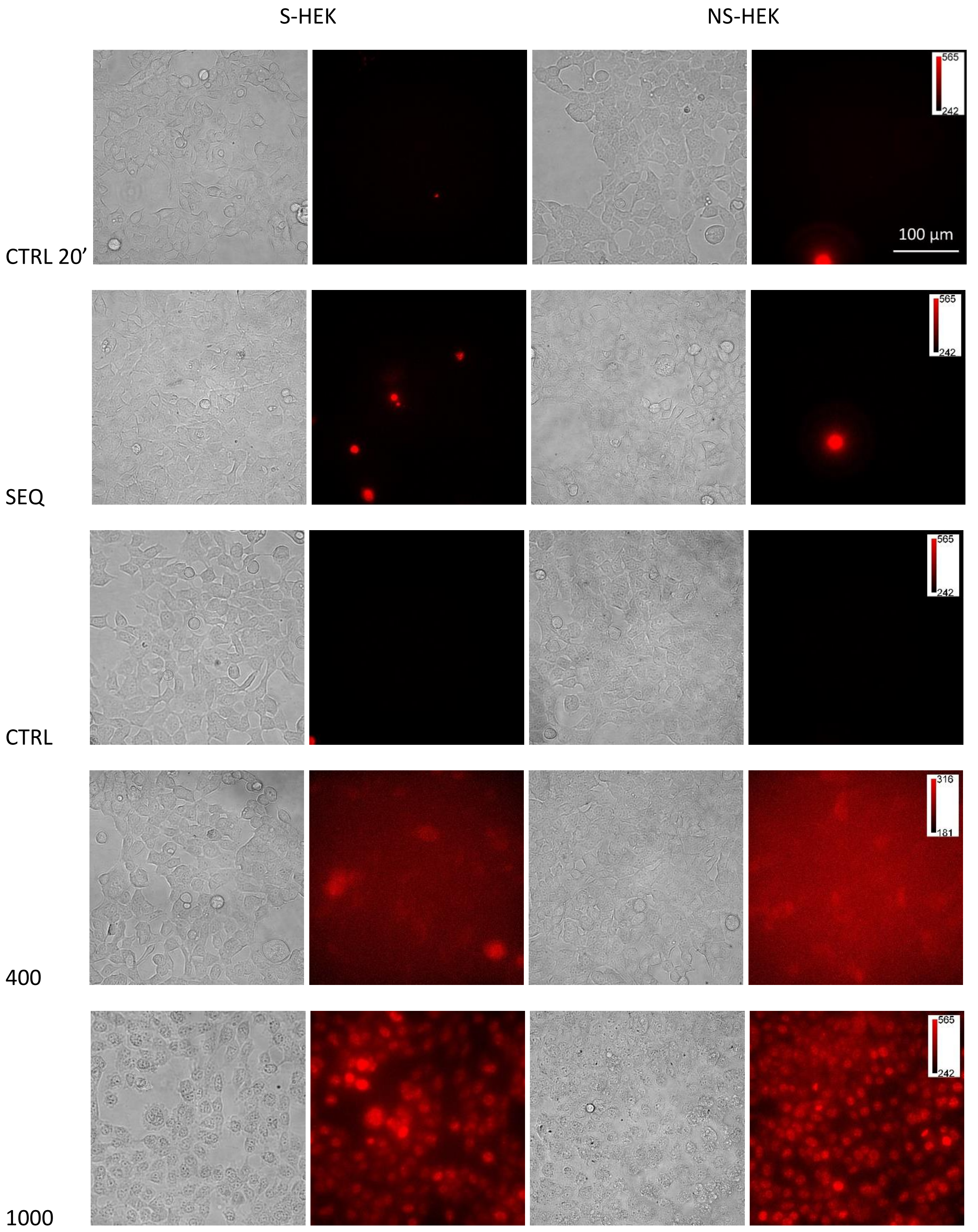

Figure S2: Propidium uptake after electric pulse delivery in excitable S-HEK and non-excitable NS-HEK cells. Representative brightfield and fluorescence images of S-HEK (left) and NS-HEK cells (right) are shown. Pulses delivered were a sequence of eight single pulses of increasing electric field 126–400 V/cm (as in ElectroFluor630 experiments, see Fig. 2a in the main part of the paper), delivered 2 min apart (denoted here as protocol SEQ), 8x 100 µs, 400 V/cm, 1 Hz (denoted as 400), and 8x 100 µs, 1000 V/cm, 1 Hz (denoted as 1000). Two controls with no pulse were performed: CTRL 20' that was checked for propidium uptake after 20 min of incubation with propidium (and served as a control for protocol SEQ) and CTRL was checked for propidium uptake after 5 min (and served as a control for protocols 400 and 1000). Note different brightness and contrast ranges to show detailed labelling of cells and nuclei with propidium (see brightness calibration bars for each corresponding line of images).

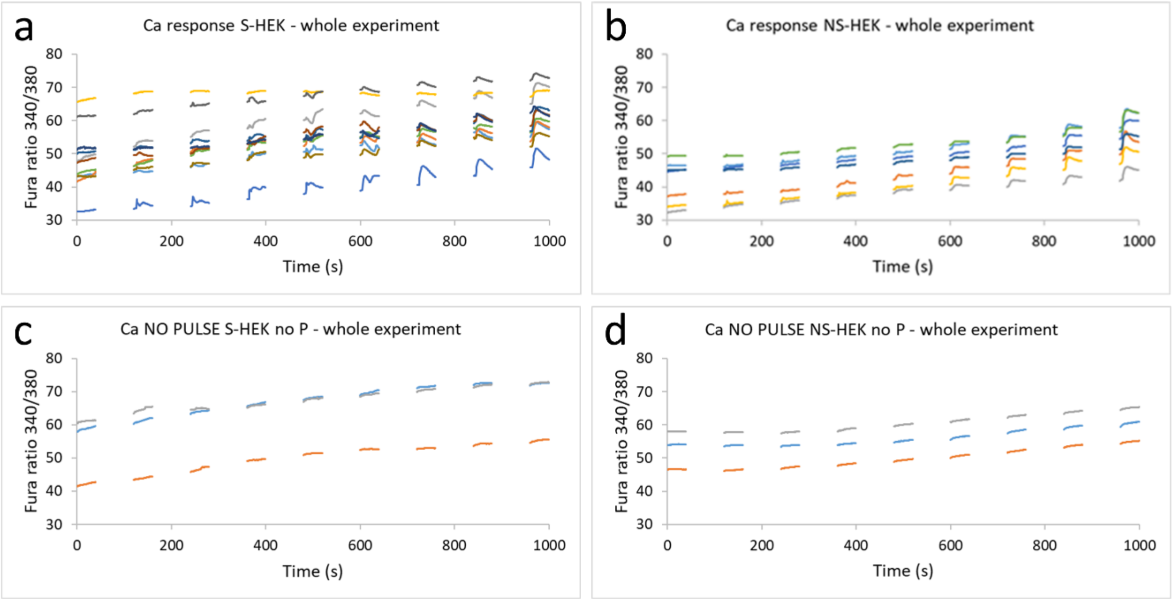

Figure S3: A slow rise of Fura-2 signal baseline throughout the duration of the experiment can be observed. Fura-2 ratio signals of all the experiments are plotted on a timescale of the whole experiment. Fura ratio in a) pulsed S-HEK cells (number of experiments: n=11), b) pulsed NS-HEK (n=7), c) S-HEK cells, not exposed to electric pulses (n=3), and d) NS-HEK cells, not exposed to electric pulses (n=3).

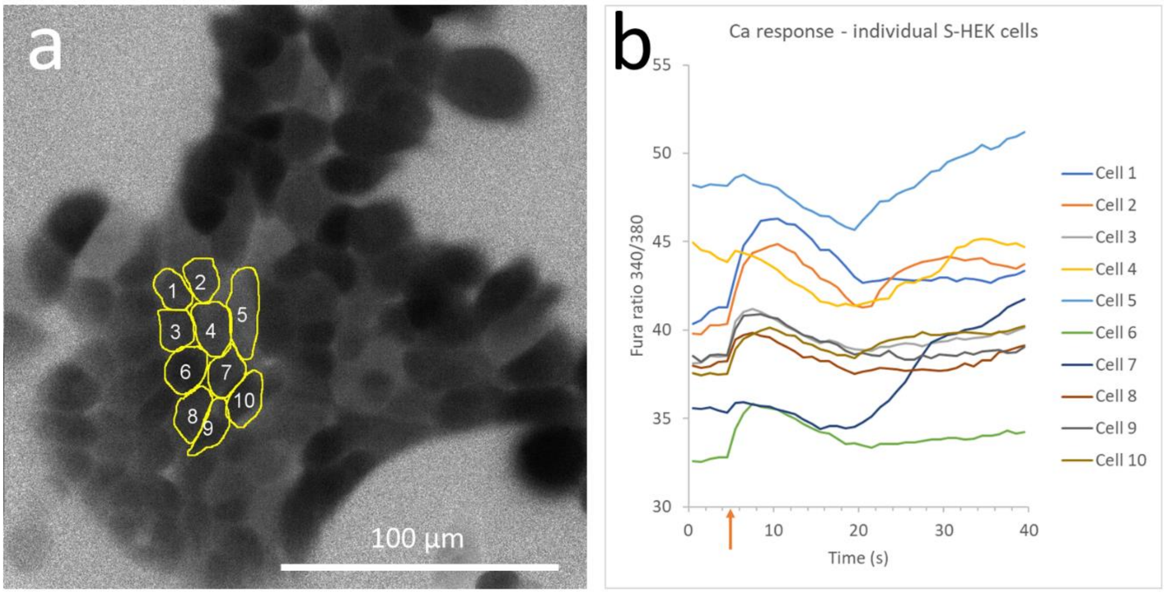

Figure S4: An uptake of Ca^{2+} ions in ten individual S-HEK cells exposed to a single 100 µs, 200 V/cm electric pulse (as a part of a sequence, see Fig. 2a and Materials and methods in the main part of the paper) as determined by Fura-2 ratio 340/380. Individual S-HEK cells were encircled in Fura ratio images (a) and average Fura-2 ratio was determined for each cell (b). The pulse was delivered at around 5 s after the image acquisition started (indicated by a red arrow).

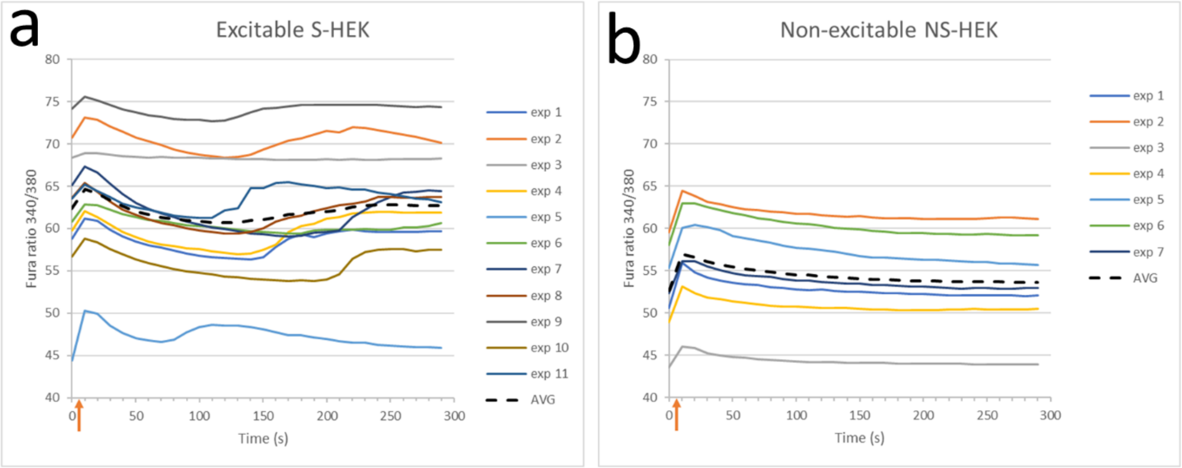

Figure S5: An uptake of Ca^{2+} ions in excitable S-HEK (a) and non-excitable NS-HEK cells (b) exposed to a single 100 µs electric pulse of 400 V/cm applied 5 min after the whole experiment (see Fig. 2a in the main part of the paper), as determined by Fura ratio 340/380. Image acquisition was done every 10 s for 5 min. Fura ratio was averaged for a whole image of each experiment. Number of experiments: N=11 for S-HEK cells and N=7 for NS-HEK cells. AVG: The average Fura ratio 340/380 of all experiments. The pulse was delivered 5 s after the image acquisition started (indicated by a red arrow).

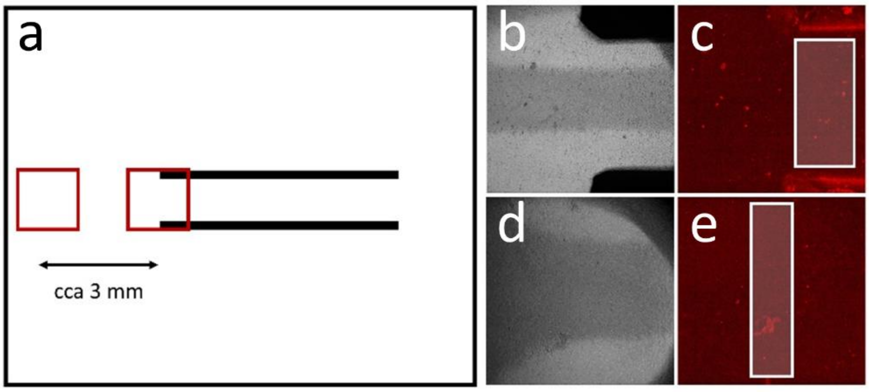

AP propagation velocity estimations (mm/s)
45.5
31.9
34
29.5
28.3
Average 33.8

Figure S6: A rough estimation of action potential (AP) propagation velocity. a: The position of two fields of view (5x objective) where the distance of AP propagation *d* was measured (cca 3 mm), right: at the ends of the electrodes, left: away from the electrodes. The schematic is not in precise ratios. b, c: An image of field of view near the electrodes, brightfield (b) and ElectroFluor630 fluorescence (c). d, e: An image of field of view away from the electrodes where the AP was recorded and the time *t* between pulse delivery and when AP was determined, brightfield (d) and ElectroFluor630 fluorescence (e). A table: Rough estimations of AP propagation velocity determined by *d*/*t*. Results from five experiments performed on three batches of cells are presented and averaged.

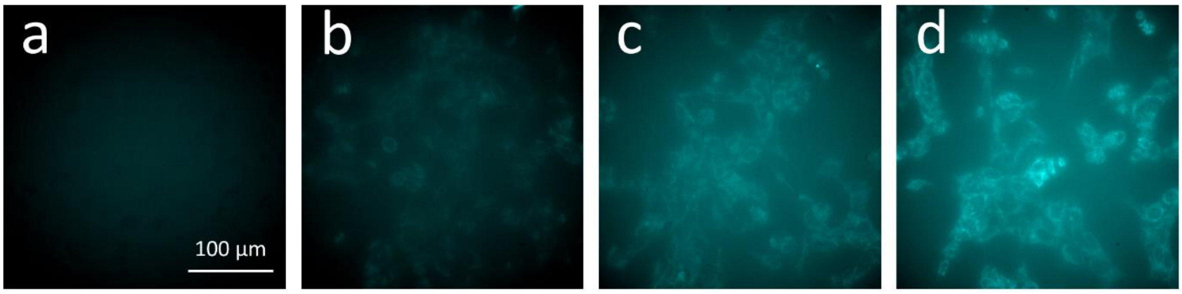

Figure S7: A doxycycline-induced, dose-dependent expression of $K_{ir}2.1$ channels in genetically modified HEK cells was confirmed by detecting a Cyan Fluorescent Protein that is co-expressed together with $K_{ir}2.1$ channels. a: No doxycycline (DOX), NS-HEK (non-spiking, non-excitable) cells, b: 2 µg/ml DOX, c: 3 µg/ml DOX, d: 4 µg/ml DOX, as used in experiments for generating S-HEK (spiking, excitable) cells. DOX was added to cells two or three days prior to the experiments (here two days DOX incubation). Cells were observed under Thunder Leica microscope, with 40× objective, 390 nm excitation wavelength and an appropriate filter set (DFT51010) at 519 nm emission.

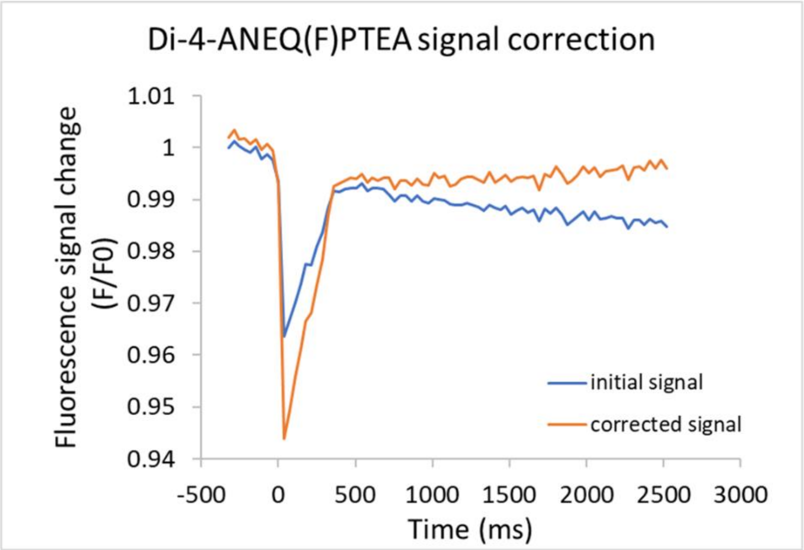

Figure S8: ElectroFluor630 (Di-4-ANEQ(F)PTEA) signal correction with a custom Matlab algorithm. A pulse was delivered at time zero. The initial signal (blue) from ElectroFluor630 images (the whole image was analysed) was weak and showed a substantial fading due to bleaching. Therefore, it was corrected with a custom Matlab algorithm (red) where by thresholding, only membranes were considered for analysis, and the signal was corrected for fluorescence fading. Furthermore, with the use of the Matlab algorithm, the peak intensity changes were detected, and time parameters indicative of dynamics of response of the signal were extracted.

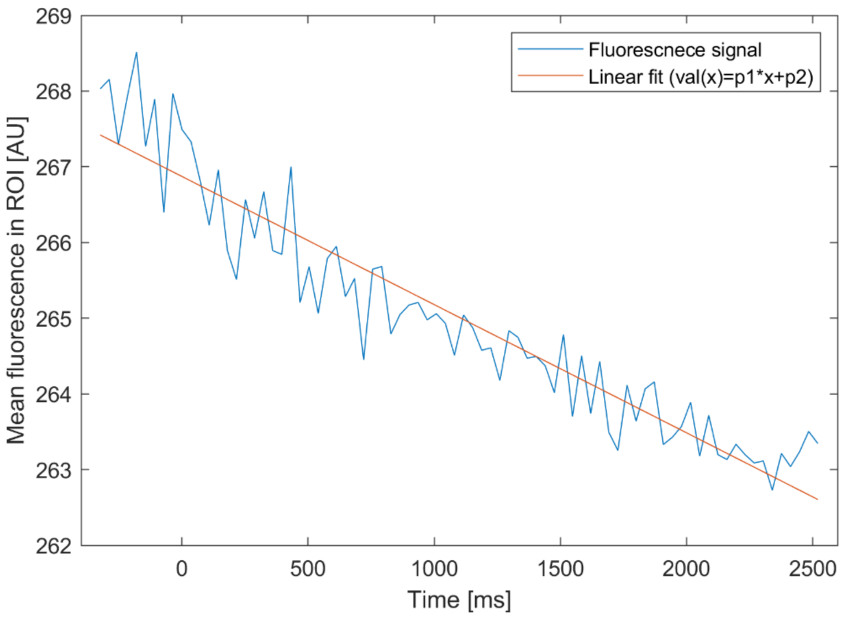

Figure S9: Illustration of the fading function. The fluorescence signal was approximated by a linear decrease (R^2=0.89). The linear fit showed an approximation of the fading function, and enabled the automatic correction of fading, by fitting the slope in the control exposure for each experiment. The function was then adjusted by setting the intercept (p2) to the mean value of the fluorescence signal before the pulses.

Table S1: Transmembrane voltage (TMV) responses of S-HEK and NS-HEK cells to electric pulses with lower amplitudes than the first experimental series (see paper, Fig. 3). Cells were exposed to three consecutive pulses of 100 µs and electric field of 40−100 V/cm, two min apart.

E (V/cm)	Cells	Nr. of experiments	Nr. of responses	Responses out of all experiments (%)	Nr. of 1 AP responses	Nr. of multiple AP responses
40	S-HEK	11	1	9.1	1	0
	NS-HEK	16	0	0.0	0	0
66	S-HEK	11	1	9.1	0	1
	NS-HEK	16	0	0.0	0	0
100	S-HEK	11	2	18.2	0	2
	NS-HEK	16	2	12.5	2	0
