# Supplementary material for: Genetically engineered HEK cells as a valuable tool for studying electroporation in excitable cells
Source: Sci Rep. 2024 Jan 6;14:720. doi: 10.1038/s41598-023-51073-5 (PMC10771480; doi:10.1038/s41598-023-51073-5)
Supplement: Supplementary file 2 — Supplementary Information 2. [file 41598_2023_51073_MOESM2_ESM.pdf]

Genetically engineered HEK cells as a valuable tool for studying electroporation in excitable cells

Tina Batista Napotnik, Bor Kos, Tomaž Jarm, Damijan Miklavčič, Rodney P. O'Connor, Lea Rems

Supplement 2

Here we provide additional information regarding computational modeling.

S1 Model of S-HEK cell array

S1.1 Voltage-dependent characteristics of $Na_V1.5$ and $K_{ir}2.1$ channels

In the model of S-HEK cells we used equations describing the currents through $Na_V1.5$ and $K_{ir}2.1$ channels from the model of ten Tusscher et al.[1]

For $Na_V1.5$:

$$m_\infty = \frac{1}{\left(1+\exp\left(-\frac{u+56.86}{9.03}\right)\right)^2} \quad \text{(S1)}$$

$$\tau_m = \alpha_m \beta_m \quad \text{(S2)}$$

$$\alpha_m = 1\ \text{ms}^{-1} \frac{1}{1+\exp\left(-\frac{u+60}{5}\right)} \quad \text{(S3)}$$

$$\beta_m = 0.1\ \text{ms}^{-1} \left(\frac{1}{1+\exp\left(\frac{u+35}{5}\right)} + \frac{1}{1+\exp\left(\frac{u-50}{200}\right)}\right) \quad \text{(S4)}$$

$$h_\infty = \frac{1}{\left(1+\exp\left(\frac{u+71.55}{7.43}\right)\right)^2} \quad \text{(S5)}$$

$$\tau_h = \frac{1}{\alpha_h+\beta_h} \quad \text{(S6)}$$

$$\alpha_h = \begin{cases} 0\ \text{ms}^{-1}, & u \geq -40 \\ 0.057\ \text{ms}^{-1} \exp\left(-\frac{u-80}{6.8}\right), & u < -40 \end{cases} \quad \text{(S7)}$$

$$\beta_h = \begin{cases} 0.77\ \text{ms}^{-1} \frac{1}{0.13\left(1+\exp\left(-\frac{u+10.66}{11.1}\right)\right)}, & u \geq -40 \\ 2.7\ \text{ms}^{-1} \exp(0.079u) + 3.1 \cdot 10^5 \exp(0.3485u), & u < -40 \end{cases} \quad \text{(S8)}$$

$$j_\infty = h_\infty \quad \text{(S9)}$$

$$\tau_j = \frac{1}{\alpha_j+\beta_j} \quad \text{(S10)}$$

$$\alpha_j = \begin{cases} 0\ \text{ms}^{-1}, & u \geq -40 \\ 1\ \text{ms}^{-1}\frac{\left(-2.5428\cdot 10^{4}\exp(0.2444u) - 6.948\cdot 10^{-6}\exp(-0.04391u)\right)(u+37.78)}{(1+\exp(0.311(u+79.23)))}, & u < -40 \end{cases} \quad \text{(S11)}$$

$$\beta_j = \begin{cases} 0.6\ \text{ms}^{-1}\frac{\exp(0.057u)}{\left(1+\exp\left(-\frac{u+32}{10}\right)\right)}, & u \geq -40 \\ 0.02424\ \text{ms}^{-1}\frac{\exp(-0.01052u)}{(1+\exp(-0.1378(u+40.14)))}, & u < -40 \end{cases} \quad \text{(S12)}$$

For $K_{ir}2.1$:

$$n_\infty = 100\frac{\alpha_n}{\alpha_n+\beta_n} \quad \text{(S13)}$$

$$\alpha_n = 0.1\ \text{ms}^{-1}\frac{1}{1+\exp(0.06(u-e_K-200))} \quad \text{(S14)}$$

$$\beta_n = 1\ \text{ms}^{-1}\frac{3\exp(0.0002(u-e_K+100)) + \exp(0.1(u-e_K-10))}{(1+\exp(-0.5(u-e_K)))} \quad \text{(S15)}$$

The factor 100 in the expression for n_∞ was added by McNamara et al.[2] to make n_∞ of order unity between −90 and −60 mV.

In eqs. (S1–S15), u is the nondimensionalized transmembrane voltage, denoted in eqs. by U_m:

$$u = \frac{U_m}{1\ \text{mV}} \quad \text{(S16)}$$

whereas e_K is a nondimensionalized reversal potential for potassium ions:

$$e_K = \frac{E_K}{1\ \text{mV}} \quad \text{(S17)}$$

S1.2 Increase in membrane conductance due to electroporation g_{ep}

To describe the increase in membrane conductance due to electroporation g_{ep} we adapted an empirical expression developed by Ivorra et al.[3] based on measurements of changes in the conductance of dense cell suspensions. The original expression is:

$$\sigma_{ep} = K\left(e^{\beta|U_m|} - 1\right) \quad \text{(S18)}$$

where the parameters K and β were fitted to experimental data. The membrane conductivity σ_{ep} (in S/m) is related to membrane conductance g_{ep} (in S/m^2) through the product:

$$\sigma_{ep} = g_{ep}d_m \quad \text{(S19)}$$

where d_m is the membrane thickness. Eq. (S18) was fitted to measurements obtained *during* the application of a high-voltage pulse. Various studies have shown that the greatest increase in membrane conductance is observed during the pulse (up to the order of 100000 S/m^2).[4] After the end of the pulse, conductance decreases by more than an order of magnitude on a microsecond time scale; however, the membrane conductance remains increased for second to minutes.[4–7] The measured values of membrane conductance on this time scale range from the order of 0.1 to 100 S/m^2.[8]

Since AP generation and propagation occur on millisecond to second time scale, they are mainly affected by the increase in membrane conductance over this time scale. Eq. (S18) does not consider the recovery of the membrane conductance after the pulse and, moreover, the kinetics of this recovery in S-HEK cells is not yet known. Based on available data from other cell types,[4–7] we assumed that on the ms−s time scale after the pulse, the membrane conductance decreases by about three orders of magnitude compared to the maximum value established during the pulse. Thus, for our calculations, we multiplied the parameter K in eq. (S18) with a factor of 0.001 and we described the kinetics of membrane recovery such, that we took the solution for g_{ep} at the end of the pulse and let g_{ep} recover exponentially with arbitrary characteristic time τ_r. As such, we obtained eq. (10) of the main manuscript:

$$g_{ep} = \begin{cases} \alpha\left(e^{\beta|U_m|} - 1\right), & t \leq t_{pulse} \\ g_{ep}\left(t = t_{pulse}\right)e^{-\frac{t}{\tau_r}}, & t \geq t_{pulse} \end{cases} \quad (10)$$

where

$$\alpha = 0.001\frac{K}{d_m} \quad (S20)$$

Parameter K further depends on internal conductivity inside a pore σ_p:[3]

$$K = 5 \cdot 10^{-9}\sigma_p \quad (S21)$$

where, according to Li and Lin,[9] σ_p depends on the extracellular σ_e and intracellular σ_i conductivity:

$$\sigma_p = \frac{\sigma_e - \sigma_i}{\ln(\sigma_e/\sigma_i)} \quad (S22)$$

Parameter α in eq. (10) is thus:

$$\alpha = \frac{5 \cdot 10^{-12}}{d_m}\frac{\sigma_e - \sigma_i}{\ln(\sigma_e/\sigma_i)} \quad (S23)$$

We also attempted to describe the increase in membrane conductance using one of the most widely used electroporation models by DeBruin and Krassowska.[10] However, in this model, the equilibrium pore density already gives such high baseline membrane conductance that it results in sustained depolarization even without applying a pulse. Further development of models describing the increase in membrane conductance during and after pulse exposure is thus required in the future for more accurate simulations of the electrophysiological response of excitable cells to electroporation.

S1.3 Implementation in Comsol Multiphysics

The partial differential equation (PDE) for electric potential distribution (eq. 4 of the main manuscript) was implemented with three separate *Electric Currents* interfaces of the *AC/DC module*, one defined in the extracellular domain, one defined in every other cell in the cell array (all nonconnected cells in a chess-board pattern), and one defined in the remaining cells. The electric current density across the cell membrane (eq. 5), was defined with the *Normal Current Density* boundary condition. The electric current density across the cell contacts (eq. 11) was defined with the *Distributed Impedance* boundary condition. The ordinary differential equations (ODEs) for the gating variables *m*, *h*, and *j* of the $Na_V1.5$ channels (eq. 9) were each implemented in a separate interface *Boundary ODEs and DAEs*. The default mesh size was used for the calculations.

The system of equations was solved within a *Time Dependent Study* with *Strict* time stepping and a *Direct*, *Fully Coupled* solver *MUMPS*.

S2 Model of S-HEK cell monolayer

S2.1 Derivation of eq. (14)

When a cell is exposed to external electric field, an induced transmembrane voltage U_m establishes on the cell membrane. The analytical expression for the induced U_m in a spherical cell with radius R_{cell} in a homogenous electric field E is:[11]

$$U_m = 1.5 R_{cell} E \cos(\theta)\left(1 - \exp\left(-\frac{t}{\tau_{chg}}\right)\right) \tag{S24}$$

where θ is the angle between the electric field direction and the unit normal to the membrane surface, and τ_{chg} is the characteristic membrane charging time:

$$\tau_{chg} \approx R_{cell} C_m \left(\frac{1}{2\sigma_e} + \frac{1}{\sigma_i}\right) \tag{S25}$$

We consider that the induced U_m on the hyperpolarized cell hemisphere, which induces an inward electric current during the pulse application, is responsible for stimulating the cell to generate an AP. The associated transmembrane electric current density J_m is:

$$J_m = g_m U_m + C_m \frac{dU_m}{dt} \tag{S26}$$

where g_m is the passive membrane conductance and C_m is the membrane capacitance. Inserting eq. (S24) into eq. (S26) yields:

$$J_m = g_m 1.5 R_{cell} E \cos(\theta)\left(1 - e^{-\frac{t}{\tau_{chg}}}\right) + \frac{g_m 1.5 R_{cell} E}{\tau_{chg}} \cos(\theta)\, e^{-\frac{t}{\tau_{chg}}} =$$

$$= 1.5 R_{cell} E \left(g_m \left(1 - e^{-\frac{t}{\tau_{chg}}}\right) + \frac{C_m}{\tau_{chg}} e^{-\frac{t}{\tau_{chg}}}\right)\cos(\theta) = f(t)\cos(\theta) \tag{S27}$$

To obtain the stimulus current, we integrate eq. (S27) over the surface S corresponding to the hyperpolarized hemisphere of the cell:

$$I_{stim} = \iint_S J_m dS = f(t)\int_0^{2\pi}\int_{\pi/2}^{\pi} {R_{cell}}^2 \sin\theta \cos\theta \, d\theta \, d\varphi = -f(t)\pi {R_{cell}}^2$$

$$I_{stim} = -1.5\pi {R_{cell}}^3 E \left(g_m \left(1 - e^{-\frac{t}{\tau_{chg}}}\right) + \frac{C_m}{\tau_{chg}} e^{-\frac{t}{\tau_{chg}}}\right) \tag{S28}$$

We then divide I_{stim} with the surface area of the cell $4\pi {R_{cell}}^2$ to obtain the average stimulus current density J_{stim}:

$$J_{stim} = -\frac{1.5}{4} R_{cell} E \left(g_m \left(1 - e^{-\frac{t}{\tau_{chg}}}\right) + \frac{C_m}{\tau_{chg}} e^{-\frac{t}{\tau_{chg}}}\right) \tag{S29}$$

Since the stimulus current is present only while the external electric field is present (while the pulse is applied), we multiply eq. (S29) with two smoothed Heaviside functions $h(t)$ (Comsol function *flc1hs*), which together describe the shape of the pulse with duration t_{pulse} applied at time t = 0 s.

$$J_{stim} = -\frac{1.5}{4} R_{cell} E \left(g_m \left(1 - e^{-\frac{t}{\tau_{chg}}}\right) + \frac{C_m}{\tau_{chg}} e^{-\frac{t}{\tau_{chg}}}\right)\left(h(t) - h(t - t_{pulse})\right) \tag{S30}$$

Eq. (S30) is equal to the eq. (14) in the main manuscript, considering that $g_m \approx g_L$. Note that for simplicity, we neglected the contribution of the resting voltage to U_m.

S2.2 Derivation of eq. (15)

The derivation of eq. (15) follows an approach similar to the derivation of eq. (14). During the pulse, the increase in membrane conductance due to electroporation in an individual cell is considered to follow (see Section S1.2):

$$g_{ep} = \alpha\left(e^{\beta|U_m|} - 1\right) \tag{S31}$$

Inserting eq. (S24) into eq. (S31) yields

$$g_{ep} = \alpha\left(e^{1.5R_{cell}E\beta|\cos(\theta)|} - 1\right) = \alpha\left(e^{A|\cos(\theta)|} - 1\right) \tag{S32}$$

where we neglected the time-dependent change in g_{ep}. Integrating (S32) over the hyperpolarized hemisphere of the membrane surface S (due to symmetry, same result would be obtained when integrating over the entire membrane surface):

$$\iint_S g_{ep} dS = \int_0^{2\pi}\int_{\pi/2}^{\pi} {R_{cell}}^2 \sin\theta\, \alpha\left(e^{A|\cos(\theta)|} - 1\right) d\theta\, d\varphi =$$

$$= 2\pi {R_{cell}}^2 \alpha \int_{\pi/2}^{\pi} \sin\theta\left(e^{A|\cos(\theta)|} - 1\right) d\theta =$$

$$= 2\pi {R_{cell}}^2 \alpha \left(\int_{\pi/2}^{\pi} \sin\theta\, e^{A|\cos(\theta)|} d\theta - \int_{\pi/2}^{\pi} \sin\theta\, d\theta\right) \tag{S33}$$

The first integral can be solved *per partes* with $u = e^{A|\cos(\theta)|}$ and $dv = \sin\theta\, d\theta$. The integration of eq. (S33) yields the solution:

$$\iint_S g_{ep} dS = 2\pi {R_{cell}}^2 \alpha \left(\frac{e^A - 1}{A} - 1\right) = 2\pi {R_{cell}}^2 \alpha \left(\frac{e^{1.5R_{cell}E\beta} - 1}{1.5R_{cell}E\beta} - 1\right) \tag{S34}$$

We divide the solution with the surface area of the hemisphere $2\pi {R_{cell}}^2$ to obtain the average increase in membrane conductance at the level of the cell monolayer G_{ep}, and we further multiply the equation with the smoothed Heaviside function $h(t)$ (Comsol function *flc1hs*):

$$G_{ep} = \alpha\left(\frac{\exp(1.5R_{cell}E\beta) - 1}{1.5R_{cell}E\beta} - 1\right) h(t) \tag{S35}$$

Eq. (S35) is equal to the eq. (15) in the main manuscript. Note that for simplicity, we neglected the contribution of the resting voltage to U_m.

S2.3 Implementation in Comsol Multiphysics

The PDE for electric potential distribution (eq. 4 of the main manuscript) was implemented with the *Electric Currents* interface of the *AC/DC module*. The PDE describing AP propagation at the boundary representing the cell monolayer (eq. 12) was implemented with the *Coefficient Form Boundary PDE* interface. Zero flux boundary condition was used at the edges of the monolayer. The ODEs for the gating variables m, h, and j of the $Na_V1.5$ channels (eq. 9) were each implemented in a separate interface *Boundary ODEs and DAEs*. To obtain a smooth solution, the maximum mesh element size along the cell monolayer was set to 200 µm.

In these simulations we first calculated the electric field distribution (eq. 4) in the entire model geometry representing the Lab-Tek well with electrodes using a *Stationary* study with an *Iterative*, *Fully Coupled* solver *Conjugate gradients*. We then used this solution, specifically the electric field distribution along the cell monolayer, as input to simulations of AP generation and propagation. The latter was simulated within a *Time Dependent Study* with *Strict* time stepping and a *Direct*, *Fully Coupled* solver *MUMPS*.

S2.4 Model parameters

Table S2: Parameters used in the models of the S-HEK cells.

**Parameter**	**Symbol**	**Value**	**Ref.**
Cell radius	R_{cell}	9 µm	[12]
Extracellular conductivity	σ_e	1.5 S/m	[13]
Intracellular conductivity	σ_i	0.5 S/m	[13]
Extracellular permittivity	ε_e	80	[13]
Intracellular permittivity	ε_i	80	[13]
Cell membrane capacitance	C_m	0.01 F/m^2	[13]
Cell membrane thickness	d_m	5 nm	[13]
Characteristic membrane charging time	τ_{chg}	210 ns	eq. (S25)
Reversal potential Na	E_{Na}	75 mV	[14]
Reversal potential K	E_K	-90 mV	[2]
Reversal potential leak	E_L	-20 mV	[2]
Max conductance Na	g_{Na}	2.75 nS/pF · C_m = 27.5 S/m^2	[14]
Max conductance K	g_K	0.05 nS/pF · C_m = 0.5 S/m^2	[2]
Max conductance leak	g_L	0.005 nS/pF · C_m = 0.05 S/m^2	[15]
Conductance of gap junctions	g_{Cxn}	1000 S/m^2	[15]
Gap junction conductance at the level of cell monolayer	G_{Cxn}	32.4 nS	eq. (S10)
Internal conductance of a pore	σ_p	0.91 S/m	eq. (S22)
Parameter in g_{ep}	α	9.1·10^{-4} S/m^2	eq. (S23)
Parameter in g_{ep}	β	16 V^{-1}	[3]

The cell size (radius 9 µm, diameter 18 µm) corresponds to the average diameter of HEK cells (diameter reported in the range of 12–24 µm)[12]. Note that the chosen cell size is consistent with typical dimensions of the cells in Fig. 1 (some cells are larger, some are smaller). Other model parameters were taken from previous studies on spiking HEK cells[2,14–16] with slight adaptations. McNamara et al.[14] used g_{Na} of 1.5 nS/pF, which in their model corresponded to a maximum transient current of 3 nA at -25 mV measured in a 20 pF patch clamp recording. They used a simplified expression for the gating variable m, approximating $m = m_\infty$. We used the full expression for m and consequently had to increase g_{Na} to 2.75 nS/pF to get a maximum transient current of 3 nA at -25 mV for a cell with capacitance of 20 pF. We added a leak current with reversal potential of -20 mV. The latter was chosen as the resting voltage of wild type HEK cells without K_{ir} (and Na_V) channels.[16] The combination of g_K and g_L was chosen such to result in an AP with full-width at half-maximum close to the one in our experiments at the lowest applied electric fields: 380 ± 145 ms (mean ± SD, N = 10), median 351 ms. Note that g_K of 0.05 nS/pF is between the values reported in McNamara et al.[2] (0–0.5 nS/pF), whereas g_L of 0.005 nS/pF is the same as used in Ori et al.[15]

The conductance of gap junctions was estimated in Ori et al.[15] to be 10 nS/pF, which for an average HEK cell with capacitance of 10 pF corresponds to 100 nS. The membrane area with which a cell in our cell array model was connected with other cells was ~1·10^{-10} m^2. Thus, we set g_{Cxn} to 1000 S/m^2.

S3 Model of a neuronal membrane

In the equivalent circuit model of a neuronal membrane, the membrane was represented with a capacitor and three resistors representing the ionic currents flowing through sodium, potassium and other (leak) channels. We used a model that was fitted to measurements on pyramidal cortical neurons.[17] We also added a resistor representing a nonselective increase in ionic current due to electroporation. The transmembrane voltage U_m was computed by solving the differential equation:

$$\frac{dU_m}{dt} = -\frac{1}{C_m}\left(I_{Na} + I_K + I_L + I_{ep} + I_{stim}\right) \tag{S36}$$

$$\frac{dU_m}{dt} = -\frac{1}{C_m}\left(g_{Na}m^3h(U_m - E_{Na}) + g_K n^4(U_m - E_K) + g_L(U_m - E_L) + g_{ep}U_m + I_{stim}\right)$$

where, similarly as in eqs. (6–8), g_X (X = Na, K, or L) is the maximal conductance of the given type of ion channel and E_X is the reversal potential for the given type of ions. g_{ep} is the nonspecific increase in membrane conductance due to electroporation, and I_{stim} is the stimulus (injected inward current). We considered that membrane conductance increases from 0 S/m^2 to a given value g_{ep} at time t = 0 s by turning on a switch (see Fig. 6h). The gating variables m, h, and n were computed by solving eq. (9).

y_∞ and τ_y (y = m, h, or n) were expressed in terms of α_y and β_y, where the latter can be understood as transition rates between the seperate states of the channels.

$$y_\infty = \frac{\alpha_y}{\alpha_y + \beta_y} \tag{S37}$$

$$\tau_y = \frac{1}{\alpha_y + \beta_y} \tag{S38}$$

$$\alpha_m = 0.182\ \mathrm{ms}^{-1}\frac{u+35}{1-\exp\left(-\frac{u+35}{9}\right)} \tag{S39}$$

$$\beta_m = -0.124\ \mathrm{ms}^{-1}\frac{u+35}{1-\exp\left(-\frac{u+35}{9}\right)} \tag{S40}$$

$$\alpha_h = 0.250\ \mathrm{ms}^{-1}\exp\left(-\frac{u+90}{12}\right) \tag{S41}$$

$$\beta_h = 0.250\ \mathrm{ms}^{-1}\frac{\exp\left(\frac{u+62}{6}\right)}{\exp\left(\frac{u+90}{12}\right)} \tag{S42}$$

$$\alpha_n = 0.020\ \mathrm{ms}^{-1}\frac{u-25}{1-\exp\left(-\frac{u-25}{9}\right)} \tag{S43}$$

$$\beta_n = -0.002\ \mathrm{ms}^{-1}\frac{u-25}{1-\exp\left(-\frac{u-25}{9}\right)} \tag{S44}$$

Here, u is again the nondimensionalized U_m, as given in (S16).

Table S3: Parameters used in the model of the neuronal membrane.

Parameter	Symbol	Value	Ref.
Reversal potential Na	E_{Na}	55 mV	[17]
Reversal potential K	E_K	-77 mV	[17]
Reversal potential leak	E_L	-65 mV	[17]
Max conductance Na	g_{Na}	400 S/m^2	[17]
Max conductance K	g_K	350 S/m^2	[17]
Max conductance leak	g_L	3 S/m^2	[17]
Membrane capacitance	C_m	0.01 F/m^2	[17]
Increase in membrane conductance due to electroporation	g_{ep}	0−100 S/m^2	arbitrary

References

1. ten Tusscher, K. H. W. J., Noble, D., Noble, P. J. & Panfilov, A. V. A model for human ventricular tissue. *Am. J. Physiol. - Heart Circ. Physiol.* **286**, H1573–H1589 (2004).
2. McNamara, H. M. *et al.* Bioelectrical domain walls in homogeneous tissues. *Nat. Phys.* **16**, 357–364 (2020).
3. Ivorra, A., Villemejane, J. & Mir, L. M. Electrical modeling of the influence of medium conductivity on electroporation. *Phys. Chem. Chem. Phys.* **12**, 10055–10064 (2010).
4. Hibino, M., Itoh, H. & Kinosita, K. Time courses of cell electroporation as revealed by submicrosecond imaging of transmembrane potential. *Biophys J* **64**, 1789–1800 (1993).
5. Pavlin, M. & Miklavčič, D. Theoretical and experimental analysis of conductivity, ion diffusion and molecular transport during cell electroporation — Relation between short-lived and long-lived pores. *Bioelectrochemistry* **74**, 38–46 (2008).
6. Tung, L., Tovar, O., Neunlist, M., Jain, S. K. & O'neill, R. J. Effects of Strong Electrical Shock on Cardiac Muscle Tissue. *Annals of the New York Academy of Sciences* **720**, 160–175 (1994).
7. Wegner, L. H., Frey, W. & Silve, A. Electroporation of DC-3F Cells Is a Dual Process. *Biophysical Journal* **108**, 1660–1671 (2015).
8. Rems, L., Viano, M., Kasimova, M. A., Miklavčič, D. & Tarek, M. The contribution of lipid peroxidation to membrane permeability in electropermeabilization: A molecular dynamics study. *Bioelectrochemistry* **125**, 46–57 (2019).
9. Li, J. & Lin, H. The current-voltage relation for electropores with conductivity gradients. *Biomicrofluidics* **4**, 013206 (2010).
10. DeBruin, K. A. & Krassowska, W. Modeling electroporation in a single cell. I. Effects of field strength and rest potential. *Biophys. J.* **77**, 1213–1224 (1999).
11. Kotnik, T., Bobanović, F. & Miklavčič, D. Sensitivity of transmembrane voltage induced by applied electric fields—A theoretical analysis. *Bioelectrochemistry and Bioenergetics* **43**, 285–291 (1997).
12. Bandmann, V. *et al.* Membrane capacitance recordings resolve dynamics and complexity of receptor-mediated endocytosis in Wnt signalling. *Sci Rep* **9**, 12999 (2019).
13. Vindiš, T. *et al.* Gene electrotransfer into mammalian cells using commercial cell culture inserts with porous substrate. *Pharmaceutics* **14**, 1959 (2022).
14. McNamara, H. M. *et al.* Geometry-Dependent Arrhythmias in Electrically Excitable Tissues. *Cell Systems* **7**, 359-370.e6 (2018).
15. Ori, H. *et al.* Observation of topological action potentials in engineered tissues. *Nat. Phys.* (2022) doi:10.1038/s41567-022-01853-z.
16. McNamara, H. M., Zhang, H., Werley, C. A. & Cohen, A. E. Optically controlled oscillators in an engineered bioelectric tissue. *Phys. Rev. X* **6**, 031001 (2016).
17. Gerstner, W., Kistler, W. M., Naud, R. & Paninski, L. *Neuronal Dynamics: From Single Neurons to Networks and Models of Cognition*. (Cambridge University Press, 2014).
